# Comparative effectiveness and safety of dual antiplatelet therapy with ticagrelor vs. clopidogrel in older adults with acute coronary syndrome: a target trial emulation study

**DOI:** 10.1093/ehjcvp/pvaf035

**Published:** 2025-05-14

**Authors:** Carole A Marxer, Paul Hjemdahl, Juan J Carrero, Edouard L Fu

**Affiliations:** Department of Medical Epidemiology and Biostatistics, Karolinska Institutet, Nobels väg 12A, SE-171 77 Stockholm, Sweden; Department of Medicine Solna, Clinical Epidemiology/Clinical Pharmacology, Karolinska Institutet and Karolinska University Hospital, SE-171 76 Stockholm, Sweden; Department of Medical Epidemiology and Biostatistics, Karolinska Institutet, Nobels väg 12A, SE-171 77 Stockholm, Sweden; Department of Clinical Sciences, Danderyd Hospital, Karolinska Institutet, SE-182 88 Stockholm, Sweden; Department of Medical Epidemiology and Biostatistics, Karolinska Institutet, Nobels väg 12A, SE-171 77 Stockholm, Sweden; Department of Clinical Epidemiology, Leiden University Medical Center, Albinusdreef 2, 2333 ZA Leiden, The Netherlands

**Keywords:** Dual antiplatelet therapy, Clopidogrel, Ticagrelor, Acute coronary syndrome, MACE, Bleeding

## Abstract

**Aims:**

There is conflicting trial evidence on the comparative effects of dual antiplatelet therapy (DAPT) with ticagrelor vs. clopidogrel in older patients with acute coronary syndromes (ACS). We aimed to assess the risk of major adverse cardiovascular events (MACE) and major bleeding in ACS patients ≥75 years initiating ticagrelor vs. clopidogrel treatment.

**Methods and results:**

We used healthcare data from the Stockholm region (2011–2021) to emulate a hypothetical target trial comparing ticagrelor vs. clopidogrel. MACE was defined as a composite of cardiovascular death, myocardial infarction, or stroke. Patients were followed for 12 months. Inverse probability of treatment weighting was used to adjust for 46 baseline confounders. We used weighted Cox proportional hazards regression to estimate hazard ratios (HRs) with 95% confidence intervals (CIs) and the weighted Aalen–Johansen estimator to estimate absolute risks (AR).

Among 4637 older patients [median age, 81 years (IQR, 77–85)], 49% initiated DAPT with ticagrelor and 51% with clopidogrel. After weighting, all confounders were balanced. Ticagrelor was associated with a lower one-year MACE risk than clopidogrel (11.1% vs. 14.9%), corresponding to an AR difference of −3.8% (95%CI, −6.8, −0.8). The HR for ticagrelor vs. clopidogrel was 0.73 (95%CI, 0.56–0.95). There was no difference in the risk of major bleeding with one-year absolute risks of 4.3% with ticagrelor vs. 4.8% with clopidogrel, and a HR of 0.89 (95%CI, 0.63–1.27).

**Conclusion:**

In ACS patients aged ≥75 years, ticagrelor was associated with a lower risk of MACE than clopidogrel. There were no differences in major bleeding, although the confidence interval was wide.

## Introduction

Dual antiplatelet therapy (DAPT), consisting of low-dose aspirin and a P2Y_12_ inhibitor, is a cornerstone in the prevention of recurrent thrombotic events in patients with an acute coronary syndrome (ACS).^1^ Given that approximately 30–40% of patients hospitalized with ACS are 75 years or older,^2,3^ and that old age is associated with increased risks of both ischaemic events and bleeding, balancing the risks and benefits of antithrombotic therapy is particularly important for this age segment.^4^

The 2023 European Society of Cardiology (ESC) guidelines recommend ticagrelor over clopidogrel for patients with ACS,^5^ based on the pivotal PLATO (PLATelet inhibition and patient Outcomes) trial.^6^ However, there is conflicting trial evidence among older patients, which are under-represented in randomized controlled trials (RCTs). A *post-hoc* analysis of the PLATO trial among 2878 ACS patients aged ≥75 years found a 11% decreased rate of major adverse cardiovascular events (MACE) and no difference in major bleeding between ticagrelor and clopidogrel.^7^ On the other hand, the POPular AGE trial found fewer major and minor bleeding events with clopidogrel compared to ticagrelor among 1002 ACS patients with non-ST-elevation ≥70 years—which was not significant when restricting to major bleeding—and no difference in the risk of MACE.^8^ Hence, the 2023 ESC guidelines^5^ provide a class 2b recommendation that clopidogrel may be considered in older patients at high bleeding risk. Furthermore, a large observational study from the Swedish SWEDEHEART registry in 14 005 patients with myocardial infarction (MI) ≥ 80 years of age found a 48% increase in the risk of major bleeding and no difference in the risk of MACE.^9^

In the absence of definitive trial evidence, well-conducted observational studies may inform clinical practice on the effectiveness and safety of drugs. Advantages include their generalizability because of the inclusion of patients with multimorbidity and polypharmacy, and the inclusion of data from more recent years. We therefore aimed to conduct a population-based cohort study following the target trial emulation framework to evaluate the effectiveness and safety of DAPT with ticagrelor vs. clopidogrel on MACE and major bleeding in patients with ACS aged 75 years or older.

## Methods

### Data sources

We used data from the Stockholm CREAtinine Measurements (SCREAM) project, a healthcare utilization cohort including all adult residents in Stockholm between 2006 and 2021.^10^ At present, the period captured spans from 2006 to 2021 and follows the complete health trajectories of approximately 3.3 million individuals.^10^ SCREAM is linked to a number of National Registers and Quality Registers,^10^ with complete information on demographic data, healthcare use, diagnoses, and therapeutic/surgical procedures, and vital status. These were enriched with laboratory test data and dispensed prescriptions at Swedish pharmacies. Registries were linked and de-identified by the Swedish National Board of Welfare and are considered to have no or minimal loss to follow-up. The regional ethical review board in Stockholm approved the study (EPN 2017/793–31) and waived the need for informed consent.

### Target trial specification and emulation

Following the target trial emulation framework,^11^ we first specified the protocol of a target trial that would evaluate the comparative effectiveness and safety of DAPT with ticagrelor vs. clopidogrel in patients with ACS. Target trial emulation is a novel framework for designing and analysing observational studies,^12,13^ and has been shown to mitigate immortal time and selection biases that have affected traditional observational studies.^14-16^ The key components of the target trial and its emulation with observational data are presented below and in Supplementary material online, *Table S1*. A graphical illustration of the longitudinal study design is depicted in Supplementary material online, *Figure S1*.

### Eligibility criteria

We included all adults ≥75 years of age with a first hospitalization for ACS who initiated DAPT with ticagrelor or clopidogrel within 14 days after hospital discharge in the Stockholm Region between 1 January 2011 and 31 December 2021 (ticagrelor was introduced in Sweden in 2011). The ACS event was defined as hospitalization due to an acute MI or unstable angina as ascertained by the ICD-10 codes (primary position) issued at hospital discharge. We excluded patients who used a P2Y_12_ inhibitor prior to initiation of DAPT. We further excluded patients who used an oral anticoagulant (OAC) in the 365 days prior to or at initiation of DAPT. We excluded patients with OAC treatment because the European^5^ and local recommendations from the Drug and Therapeutics Committee favoured the use of clopidogrel over the more potent P2Y_12_ inhibitors in patients on OAC treatment. Including these patients would have created a biased setting for comparing treatments. Codes for inclusion and exclusion criteria are shown in Supplementary material online, *Table S2* and *Table S3*.

### Treatment strategies

We compared the following two treatment strategies: ‘initiate DAPT with ticagrelor and continue use for a year’ vs. ‘initiate DAPT with clopidogrel and continue use for a year’ (see Supplementary material online, *Table S4*). Initiation of DAPT was defined as a dispensation of ticagrelor or clopidogrel within 14 days after the hospital discharge date, in combination with a dispensation of low-dose aspirin within 180 days prior to or at the day of initiation of the P2Y_12_ inhibitor (accounting for the fact that some patients were already treated with aspirin for prevention of cardiovascular events). Since in-hospital drug dispensations are not recorded in our data source, we required a pharmacy dispensation of ticagrelor or clopidogrel within 14 days after the date of hospital discharge.

### Treatment assignment

Patients who filled a prescription for clopidogrel within 14 days after hospital discharge were assigned to the clopidogrel group, and patients who filled a prescription for ticagrelor within 14 days after hospital discharge were assigned to the ticagrelor group. We allowed a 14-day period after hospital discharge to claim the treatment in order to increase comparability with the results of the large Swedish SWEDEHEART study.^9^ Of note, more than 90% of the patients filled their prescriptions on the day of discharge from the hospital.

### Start and end of follow-up

Follow-up was started (i.e. time zero) on the day of treatment assignment. Patients were followed until emigration, administrative censoring (31 December 2021), the occurrence of an outcome, or 365 days of follow-up, whichever occurred first (see Supplementary material online, *Table S5*).

### Outcomes

The effectiveness outcome was MACE, defined as a composite of cardiovascular death, hospitalization for MI, and hospitalization for stroke. The safety outcome was major bleeding, defined as a composite of haemorrhagic stroke, gastrointestinal bleeding, anaemia-related bleeding, and other major bleeding requiring hospitalization. To increase comparability with bleeding results of the large Swedish SWEDEHEART study,^9^ we based our definition on the same ICD codes. Definitions are provided in Supplementary material online, *Table S6*.

### Confounders

Baseline confounders included age at DAPT initiation, sex, level of education, calendar year of DAPT initiation, type of ACS (MI vs. unstable angina), percutaneous coronary intervention (PCI) during hospitalization with ACS, medical history [diabetes mellitus, hypertension, atrial fibrillation, other arrythmias, vascular disease, chronic obstructive pulmonary disease (COPD), cancer, liver disease, heart failure, dyslipidemia, hypothyroidism, intracranial haemorrhage, valvular heart disease, bleeding, anaemia, kidney disease, alcoholism, stroke/transient ischaemic attack (TIA)/embolism, rheumatoid arthritis], medications [β-blockers, calcium channel blockers, diuretics, proton-pump inhibitors (PPIs), non-steroidal anti-inflammatory drugs (NSAIDs), lipid-lowering therapy, α-blockers, nitrate, antiarrhythmics, diabetes medications, opioids, angiotensin-converting enzyme (ACE) inhibitors, angiotensin receptor blockers (ARBs), corticosteroids, antidepressants], healthcare use in the previous year (number of unique medications, primary care visits, cardiovascular primary care visits, specialist care visits, cardiovascular specialist care visits, hospitalizations, cardiovascular hospitalizations), and laboratory measurements [estimated glomerular filtration rate (eGFR), total cholesterol, LDL cholesterol, HDL cholesterol, triglycerides). We calculated eGFR with the 2009 Chronic Kidney Disease Epidemiology Collaboration (CKD-EPI) formula without considering race.^17,18^ Covariate assessment windows and definitions of covariates are shown in Supplementary material online, *Figure S1* and Supplementary material online, *Table S7*.

### Causal estimand

In our primary analysis, we estimated an intention-to-treat (ITT) effect, i.e. the effect of treatment assignment. Thus, patients were analysed according to their original treatment assignment regardless of whether they discontinued their medication or switched to an alternative antiplatelet agent.

In secondary analyses, we estimated a per-protocol (PP) effect, i.e. the effect of following the assigned strategy. In this analysis, we censored if and when the patient did not refill his prescription for the assigned P2Y_12_ inhibitor within 90 days after the estimated end of pill supply from the previous dispensation. In this secondary analysis, we focused on the effect of continuous treatment to offer more clinically relevant treatment effect estimates, since patients may discontinue their initial treatment in clinical practice.

### Statistical analyses

Continuous variables are presented as mean with standard deviation or median with interquartile range (IQR), depending on the distribution, and categorical variables as numbers and percentages. We used inverse probability of treatment weighting (IPTW) to adjust for baseline confounding.^19,20^ We estimated the probability of initiating ticagrelor vs. clopidogrel [i.e. the propensity score (PS)] as a function of 46 baseline covariates (variables reported in footnote of *Table 2*) using a multivariable logistic regression model). Patients in the ticagrelor group were weighted by 1/PS and in the clopidogrel group by 1/(1-PS) [average treatment effect, ATE]. Weights were stabilized by using the marginal probability of the received treatment as the numerator of the weights. We calculated standardized mean differences (SMDs) to assess the balance of covariates between the treatment groups before and after weighting, using an SMD of >0.1 as the threshold for meaningful imbalance.

We used a weighted Cox proportional hazard model to estimate hazard ratios (HRs) and 95% confidence intervals (CI; robust variance estimator). More information about the choice of this method is given in the Supplementary Material (Remarks on methodology).

In the primary analysis, we additionally plotted weighted cumulative incidence curves using the Aalen–Johansen estimator, which does not overestimate absolute risks (ARs) in the presence of the competing risk of death.^21^ We calculated the 95% CI for the absolute risk (AR) using a nonparametric bootstrap with 1000 samples.

We performed pre-specified subgroup analyses stratified by sex, eGFR at baseline (<60 vs. ≥ 60 mL/min/1.73 m^2^), and presence of PCI during the ACS hospitalization. We calculated *P*-values for interaction in subgroups using a Wald test.

To determine the potential impact of unmeasured confounding (especially frailty), we conducted *post-hoc* quantitative bias analyses using the array approach.^22^

All statistical analyses were performed using R version 4.3 (R Foundation for Statistical Computing, Vienna, Austria).

## Results

### Baseline characteristics

We included 4637 older adults who survived a first-ever hospitalization for ACS, of which 2295 (49%) initiated DAPT with ticagrelor and 2342 (52%) initiated DAPT with clopidogrel within 14 days from hospital discharge (see Supplementary material online, *Figure S2*: Flow chart of cohort enrolment). The proportion of ticagrelor users, compared to clopidogrel users, increased from 2% in 2011 to 72% in 2018 and thereafter plateaued between 70% and 73% (see Supplementary material online, *Table S8*). The median age in the ticagrelor group was 79 years (IQR, 76–82) vs. 83 years (IQR, 79–88) in the clopidogrel group, and less patients were female in the ticagrelor group (36.8% vs. 50.4%). In both groups, most patients were hospitalized due to an acute MI (ticagrelor: 87.8%, clopidogrel: 88.7%), and more patients underwent PCI during the hospitalization in the ticagrelor group (88.1% vs. 49.9%). Patients in the clopidogrel group had more frequent histories of cardiovascular and other comorbid conditions, used more medications, had more recent encounters with the healthcare system, and more often had an eGFR ≤60 mL/min/1.73 m^2^ (33.6% vs. 21.0%). Numbers and proportions of main baseline characteristics are reported in *Table 1*, and all baseline characteristics are presented in Supplementary material online, *Table S9*. After weighting, all covariates were well balanced between treatment strategies (*Table 1*; Supplementary material online, *Table S8*). Further details with regard to IPTW are shown in Supplementary material online, *Figure S3*, *Figure S4*, and Supplementary material online, *Table S10*.

**Table 1:** Selected baseline characteristics of the study population overall and by treatment assignment before and after inverse probability treatment weighting (IPTW)

	Before IPTW			After IPTW		
	Ticagrelor	Clopidogrel	SMD^d^	Ticagrelor	Clopidogrel	SMD^d^
Number of individuals	2295	2342		2256	2326	
Age [years], median (IQR)	79 [76, 82]	83 [79, 88]	0.798	80 [77, 85]	81 [77, 85]	0.049
Age category [years]						
75–80	1297 (56.5)	684 (29.2)	0.574	999 (44.3)	991 (42.6)	0.034
80–85	725 (31.6)	659 (28.1)	0.077	686 (30.4)	694 (29.8)	0.013
≥85	273 (11.9)	999 (42.7)	0.737	572 (25.3)	641 (27.6)	0.052
Female sex	845 (36.8)	1180 (50.4)	0.276	989 (43.8)	1018 (43.8)	0.001
Comorbidities^b^						
Hypertension	1760 (76.7)	1849 (78.9)	0.054	1732 (76.8)	1798 (77.3)	0.013
Diabetes mellitus	577 (25.1)	621 (26.5)	0.031	587 (26.0)	602 (25.9)	0.003
Heart failure	455 (19.8)	842 (36.0)	0.366	568 (25.2)	657 (28.3)	0.070
Valvular heart disease	120 (5.2)	265 (11.3)	0.222	184 (8.2)	194 (8.3)	0.006
Atrial fibrillation	110 (4.8)	374 (16.0)	0.373	265 (11.7)	255 (11.0)	0.024
Other arrythmia	308 (13.4)	416 (17.8)	0.120	353 (15.6)	358 (15.4)	0.006
Stroke/TIA/embolism	230 (10.0)	401 (17.1)	0.208	275 (12.2)	318 (13.7)	0.044
Intracranial haemorrhage	3 (0.1)	11 (0.5)	0.062	4 (0.2)	7 (0.3)	0.023
Bleeding	186 (8.1)	301 (12.9)	0.155	219 (9.7)	249 (10.7)	0.033
Dyslipidemia	971 (42.3)	809 (34.5)	0.160	888 (39.4)	921 (39.6)	0.005
Co-medications^c^						
β-Blockers	1919 (83.6)	2009 (85.8)	0.060	1946 (86.3)	1980 (85.1)	0.032
ACE inhibitors	1215 (52.9)	1177 (50.3)	0.054	1167 (51.7)	1210 (52.0)	0.006
Angiotensin II receptor blockers	842 (36.7)	707 (30.2)	0.138	773 (34.3)	758 (32.6)	0.036
Calcium channel blockers	862 (37.6)	846 (36.1)	0.030	869 (38.5)	877 (37.7)	0.017
Diuretics	596 (26.0)	987 (42.1)	0.346	714 (31.6)	805 (34.6)	0.063
Statins	2160 (94.1)	1852 (79.1)	0.453	1987 (88.1)	2015 (86.6)	0.044
Nitrate	1876 (81.7)	1724 (73.6)	0.196	1763 (78.1)	1791 (77.0)	0.028
Diabetes medication	433 (18.9)	436 (18.6)	0.006	425 (18.8)	439 (18.9)	0.001
Laboratory measurements^c^						
eGFR ≤ 60 mL/min/1.73 m²^a^	481 (21.0)	786 (33.6)	0.286	583 (25.9)	674 (29.0)	0.070
High total cholesterol (≥ 5 mmol/L)^a^	987 (43.0)	804 (34.3)	0.179	869 (38.5)	883 (38.0)	0.010
High LDL cholesterol (≥ 4 mmol/L)^a^	336 (14.6)	257 (11.0)	0.108	306 (13.6)	297 (12.7)	0.027
Low HDL cholesterol (< 1.0 mmol/L in men, < 1.2 mmol/L in women)^a^	419 (18.3)	420 (17.9)	0.010	384 (17.0)	449 (19.3)	0.060
High triglycerides (≥ 2.26 mmol/L)^a^	178 (7.8)	147 (6.3)	0.059	156 (6.9)	162 (6.9)	<0.001

Values are *n* (%), unless otherwise indicated.

IPTW, inverse probability of treatment weighting; SMD, standardized mean difference; IQR, interquartile range; TIA, transient ischaemic attack; COPD, chronic obstructive pulmonary disease; ACE, angiotensin-converting enzyme; eGFR, estimated glomerular filtration rate; LDL, low-density lipoprotein; HDL, high-density lipoprotein.

^a^eGFR, total cholesterol, LDL cholesterol, HDL cholesterol, and triglycerides were missing in 26.5%, 14.4%, 17.5%, 17.4%, and 16.8%.

^b^Comorbidities: Any time prior to index date.

^c^Medications and laboratory measurements: 1 year prior to index date.

^d^A standardized mean difference (SMD) above 0.1 indicates a meaningful imbalance between groups.

### Primary analyses

**Figure 1:**
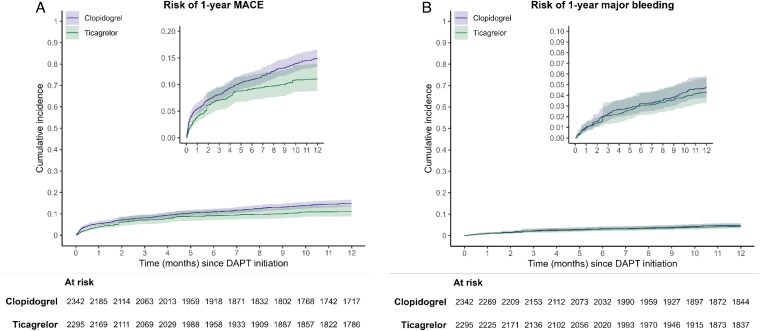
Cumulative incidence curves for one-year MACE (panel *A*) and major bleeding (panel *B*) stratiﬁed by treatment arm (DAPT with ticagrelor vs. clopidogrel), after inverse probability of treatment weighting (IPTW).

*MACE:* In total, 204 patients in the ticagrelor group and 432 patients in the clopidogrel group experienced a MACE event, corresponding to incidence rates of 10.3 and 22.3 events per 100 person-years (py), respectively (*Table 2*). Ticagrelor initiation, compared with clopidogrel initiation, was associated with a lower rate of MACE (adjusted HR, 0.73; 95% CI, 0.56–0.95; *Table 2*). Weighted cumulative incidence curves (*Figure 1*) showed a lower AR of MACE in the ticagrelor group throughout follow-up. The one-year ARs of MACE were 11.1% (95% CI, 8.8–13.9%) in the ticagrelor group and 14.9% (95% CI, 13.3–16.6%) in the clopidogrel group, resulting in a weighted AR difference of −3.8% (95% CI, −6.8 to −0.8%; *Table 2*).

**Table 2:** Number of events, incidence rates, crude, and adjusted HRs for the association of DAPT with ticagrelor vs. clopidogrel initiation and MACE and major bleeding, as well as absolute risk and absolute risk difference of the outcomes MACE and major bleeding

Outcomes	Exposure	No. of patients^a^	No. of events^a^	py^a^	IR per 100 py (95% CI)^a^	Crude HR (95% CI)	IPTW adjusted HR (95% CI)^b^	Absolute risk at 1 year, % (95% CI)	Absolute risk difference at 1 year, % (95% CI)
MACE	Clopidogrel	2342	432	1937	22.3 (20.3–24.5)	Reference	Reference	14.9 (13.3–16.6)	Reference
	Ticagrelor	2295	204	1977	10.3 (9.0–11.8)	0.47 (0.40–0.55)	0.73 (0.56–0.95)	11.1 (8.8–13.9)	−3.8 (−6.8 to -0.8)
Major bleeding	Clopidogrel	2342	116	2044	5.7 (4.7–6.8)	Reference	Reference	4.8 (3.9–5.9)	Reference
	Ticagrelor	2295	105	2037	5.2 (4.2–6.2)	0.91 (0.70–1.19)	0.89 (0.63–1.27)	4.3 (3.3–5.6)	−0.5 (−2.0 to 1.1)

no., number; py, person-years; IR, incidence rate; HR, hazard ratio; IPTW, inverse probability of treatment weighting; MACE, major adverse cardiovascular event.

^a^Number of patients, number of events, py, and IRs were calculated in the unweighted population.

^b^Analyses were adjusted through inverse probability of treatment weighting for age, sex, level of education, percutaneous coronary intervention during ACS hospitalization, type of acute coronary syndrome, medical history (diabetes mellitus, hypertension, atrial fibrillation, other arrythmias, vascular and cerebrovascular diseases (including stroke/TIA/embolism), COPD, cancer, liver disease, heart failure, dyslipidemia, hypothyroidism, intracranial haemorrhage, valvular heart disease, bleeding, anaemia, kidney disease, alcoholism, and rheumatoid arthritis), medications (β-blockers, calcium channel blockers, diuretics, NSAIDs, lipid-lowering therapy, α-blockers, nitrate, antiarrhythmics, diabetes medications, opioids, ACE inhibitors (including combinations), ARBs (including combinations), corticosteroids, antidepressants), and healthcare use in the previous year (number of unique medications, primary care visits, cardiovascular primary care visits, specialist care visits, cardiovascular specialist care visits, hospitalizations, and cardiovascular hospitalizations).

*Major bleeding:* In total, 105 patients in the ticagrelor group and 116 patients in the clopidogrel group experienced a major bleeding event, corresponding to incidence rates of 5.2 and 5.7 events per 100 py, respectively (*Table 2*). There was no statistically significant difference in the rate of major bleeding between the two groups (adjusted HR, 0.89; 95% CI, 0.63–1.27) and no difference in the cumulative incidence curves (*Figure 1*) throughout follow-up. The one-year AR of major bleeding was 4.3% for ticagrelor and 4.8% for clopidogrel with a weighted ARD of −0.5% (95% CI, −2.0 to 1.1%; *Table 2*).

### Secondary analyses

**Figure 2:**
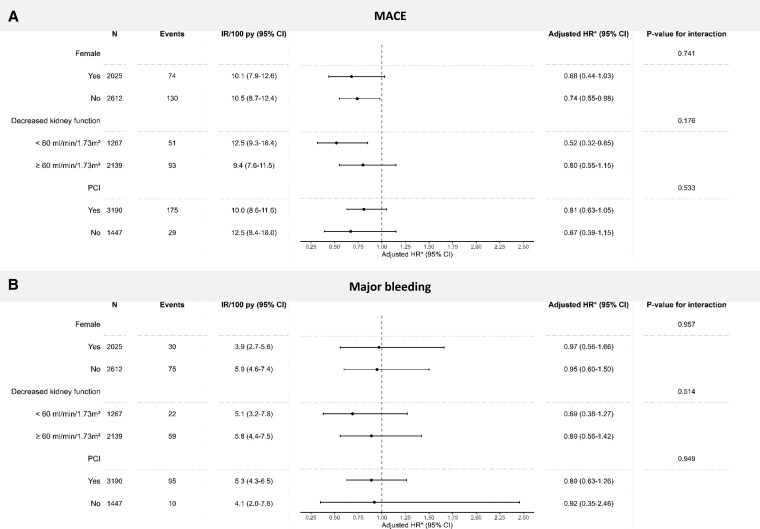
Number of events, incidence rates, and adjusted HRs for the association of DAPT with ticagrelor vs. clopidogrel initiation and MACE (panel *A*) and major bleeding (panel *B*) across subgroups.

Results were consistent in subgroups, and we did not identify effect measure modification by sex, eGFR, or PCI during index hospitalization (*Figure 2*, Supplementary material online, *Table S11*). In total, 1326 patients (28.6%) discontinued the assigned treatment strategy prematurely during the follow-up. The proportion of discontinuers was higher in the ticagrelor group than in the clopidogrel group (31.7% vs. 25.5%). In the per-protocol analysis, ticagrelor initiation, compared with clopidogrel initiation, was associated with a similarly lower rate of MACE as in the primary analysis (adjusted HR, 0.79; 95% CI, 0.59–1.07; Supplementary material online, *Table S12*). There was no difference in the rate of major bleeding between the two groups (adjusted HR, 0.96: 95% CI, 0.64–1.45; Supplementary material online, *Table S12*).

### Quantitative bias analyses

Results obtained from the *post-hoc* quantitative bias analyses are explained and shown in Supplementary material online, *Table S13*.

## Discussion

This population-based target trial emulation study among older patients with ACS showed that DAPT initiation with ticagrelor was associated with a 27% lower rate of MACE (stroke, MI, or cardiovascular death) than DAPT initiation with clopidogrel, with no significant difference for major bleeding in the ITT analysis, but the 95% CI for major bleeding was wide. Results remained when stratifying by sex, eGFR at baseline (<60 vs. ≥ 60 mL/min/1.73 m^2^), and presence of PCI during the ACS hospitalization, as well as when taking treatment discontinuation during the one-year follow-up period into account.

Major society guidelines have generally recommended the use of a higher-potency P2Y_12_ inhibitor when the bleeding risk is acceptable (2023 ECS guidelines,^5^ 2021 ACC/AHA/SCAI guidelines.^23^) Ticagrelor has been the preferred high potency agent in Sweden based on the pivotal PLATO trial in 18 624 ACS patients which showed a 16% decrease of the composite outcome of cardiovascular death, MI, or stroke and a reduced all-cause mortality among patients treated with ticagrelor compared to clopidogrel, with no significant difference in major bleeding overall.^6^ Thus, the utilization of DAPT with ticagrelor increased gradually in the Stockholm region (present study) and nationwide,^9^ as well as in other countries.^24,25^

Approximately 30%–40% of all patients hospitalized with ACS are ≥75 years old,^2,3^ which highlights the importance of studying this age segment, which is under-represented in RCTs. Old age is associated with increased risks of both ischaemic events and bleeding, and balancing the risks and benefits of antithrombotic therapy is especially important in this age segment.^4^ Our real-world findings of a 27% reduction of ischaemic events among ACS patients aged ≥75 years without a significant increase in major bleeding are in line with results from the PLATO substudy^7^ of patients aged ≥75 years but at odds with the POPular AGE trial^8^ and the large observational Swedish SWEDEHEART study.^9^

The PLATO substudy in 2878 patients found that results did not differ significantly between patients aged ≥75 vs. < 75 years.^7^ An 11% decrease in MACE with ticagrelor compared to clopidogrel among the elderly patients was not significant—likely due to limited statistical power—but cardiovascular and all-cause death remained significantly lower with ticagrelor among the elderly; there was no difference in major bleeding (HR, 1.02; CI, 0.82–1.27).^7^

The POPular AGE trial in 1002 patients ≥70 years of age with non-ST-elevation ACS reported a lower primary bleeding outcome (PLATO major or minor bleeding) with clopidogrel compared to ticagrelor (HR, 0.71; 95% CI, 0.54–0.94).^8^ The POPular AGE trial found no difference in cardiovascular death, MI, or stroke (composite outcome; HR, 0.92; 95% CI, 0.64–1.34).^8^ However, PLATO major bleeding was not significantly lower with clopidogrel compared to ticagrelor treatment (HR, 0.71; 95% CI, 0.47–1.08), and the study included patients treated with oral anticoagulants (20% in the ticagrelor group and 16% in the clopidogrel group) whereas we excluded patients who were discharged with an OAC. Furthermore, premature discontinuation of treatment was more unbalanced in the open-label POPular AGE trial (47% with ticagrelor and 22% with clopidogrel) than in our study (31.7% vs. 25.5%, respectively). Such discontinuations may reflect adverse events (e.g. dyspnoea with ticagrelor or bleeding), or intentional shortening of the treatment duration due to a high bleeding risk.

Our results also differ from those of the large SWEDEHEART register study^9^ (2010–2017) in 14 005 MI patients ≥80 years of age which found no difference between ticagrelor and clopidogrel regarding the rate of MACE (HR, 0.97; 95% CI, 0.88–1.06) but a higher risk of bleeding requiring hospitalization (HR, 1.48; 95% CI, 1.25–1.76) with ticagrelor. The authors confirmed that ticagrelor was associated with fewer MIs and strokes among patients <80 years.^9^ Our study is smaller and based on regional data for all-comers with a first hospitalization for ACS (as in the PLATO trial), whereas SWEDEHEART is a national registry. The SWEDEHEART study used a MACE with all-cause rather than cardiovascular death and had a narrower qualifying diagnosis of ACS (i.e. restriction to MI) and excluded patients with prior bleeding, which limits comparability with results from the PLATO trial^6,7^ and our (emulated trial) results. We chose a lower cut-off for old age than in the SWEDEHEART study^9^ (75 vs. 80 years) to maintain reasonable statistical power in analyses of our smaller dataset. Including 75- to 79-year-old patients in our study may have contributed to why we found a reduced risk of MACE, unlike the results of the SWEDEHEART study, but statistical power is too limited to investigate further.

Another population-based cohort study from Canada (2012–2016) reported no superiority of ticagrelor over clopidogrel in terms of MACE (HR, 0.97; 95% CI, 0.73–1.20) and a 54% increase in major bleeding (HR, 1.54; 95% CI, 1.12–2.10) in a subgroup of 1988 patients ≥75 years with ACS undergoing PCI.^25^ However, it is not reported how well balanced the elderly subgroups were, and restriction to patients with ACS who underwent PCI (vs. all ACS patients in our study and the PLATO trial) may partially explain the differences in risk estimates.^28^ An observational study from Germany (2006–2017) in 1087 patients with ST-segment–elevation MI aged ≥75 years showed similar results as in our study, with a 31% lower risk of a combined ischaemic outcome and no difference in serious bleeding events with ticagrelor vs. clopidogrel.^26^

Strengths of our study include the use of the target trial emulation framework^11^, which mitigates time-related biases such as immortal and survivor bias, and the setting of a universal tax-funded health system, which minimizes selection bias from disparate access to health care and/or reporting to a quality register. Use of the target trial emulation framework^11^ allowed us to answer our research question based on a larger population than in the previous RCTs focusing on older patients. This underscores the importance of target trial emulation studies, particularly in countries like Sweden, where healthcare data are granular. Further, our study reflects real-world clinical practice during recent years, allowing for take discontinuation of treatment into account, resulting in high generalizability of our findings, a limitation of previous trials. Lastly, we had information on laboratory measurements such as eGFR, cholesterol, and triglycerides, with comparable levels in both groups after IPTW.

The following limitations need to be acknowledged. First, although we adjusted for 46 confounders and performed quantitative bias analyses, we cannot completely rule out the possibility of residual confounding, as in all observational studies, especially residual confounding driven by unmeasured confounders (e.g. frailty, cognitive status). However, results from the quantitative bias analyses suggest that even strong, potentially unmeasured confounders like frailty are unlikely to alter our conclusions. Second, we do not know the medication behaviour of our patients once they have claimed their prescriptions. Pharmacy claims are, however, better than prescription data as they exclude patients with primary non-adherence and allow discontinuation to be taken into account in the statistical analysis. Third, our study was based on data from the Stockholm region, limiting its applicability to other populations with different healthcare systems, demographics, and treatment guidelines. However, the present real-world data are from a complete healthcare system with granular information. Similar studies in other countries would be of interest since a future RCT comparing ticagrelor and clopidogrel in elderly patients is highly unlikely. Furthermore, we did not report any data on prasugrel treatment due to its very low utilization in our region.

## Conclusion

In conclusion, this large, population-based cohort study resembling the PLATO study suggests that DAPT with ticagrelor is a good choice for DAPT among patients with ACS at 75 years of age or older as it is associated with a decreased risk of MACE with no increase in major bleeding compared to clopidogrel, also when taking treatment discontinuation during follow-up into account.

It would be of interest to perform pooled studies based on data from several countries to increase generalizability and to increase the sample size needed for further subgroup analyses, as well as to obtain more data on major bleeding, as the 95% CI for this outcome had a wide confidence interval in the present study.

## Supplementary Material

pvaf035_Supplementary_Data

## Data Availability

The data may be shared on reasonable request to Prof. Carrero (juan.jesus.carrero@ki.se) for academic research collaborations that comply with the General Data Protection Regulation, as well as national and institutional ethics regulations and standards.
