# Supplementary material for: Comparative effectiveness and safety of dual antiplatelet therapy with ticagrelor vs. clopidogrel in older adults with acute coronary syndrome: a target trial emulation study
Source: Eur Heart J Cardiovasc Pharmacother. 2025 May 14;11(6):500–8. doi: 10.1093/ehjcvp/pvaf035 (PMC12450599; doi:10.1093/ehjcvp/pvaf035)
Supplement: pvaf035_Supplementary_Data [file pvaf035_supplementary_data.docx]

SUPPLEMENTARY MATERIAL

**Title:** Comparative effectiveness and safety of dual antiplatelet therapy with ticagrelor versus clopidogrel in older adults with acute coronary syndrome: a target trial emulation study

**Authors:** Carole A. Marxer, Paul Hjemdahl, Juan J. Carrero, Edouard L. Fu

Table of Contents

[**Study protocol** 3](#_Toc194328042)

[**Protocol of the hypothetical trial and the emulation procedure** 3](#_Toc194328043)

[**Table S1:** Protocol of the hypothetical target trial and the emulation procedure. 3](#_Toc194328044)

[**Graphical overview of longitudinal study design** 5](#_Toc194328045)

[**Figure S1:** Graphical overview of longitudinal study design. 5](#_Toc194328046)

[**Eligibility criteria** 5](#_Toc194328047)

[**Table S2:** Codes used to identify hospitalizations with an acute coronary syndrome. 5](#_Toc194328048)

[**Table S3:** Codes used to identify exclusion criteria. 6](#_Toc194328049)

[**Treatment strategies** 6](#_Toc194328050)

[**Table S4:** Definitions of treatment assignment. 6](#_Toc194328051)

[**Outcomes & censoring reasons** 7](#_Toc194328052)

[**Table S5:** Definitions of censoring reasons. 7](#_Toc194328053)

[**Table S6:** Definitions of outcomes. 7](#_Toc194328054)

[**Covariates** 8](#_Toc194328055)

[**Table S7:** Definitions of covariates. 8](#_Toc194328056)

[**Remarks on methodology** 13](#_Toc194328057)

[Weighted Cox proportional hazards regression model 13](#_Toc194328058)

[**Further results** 15](#_Toc194328059)

[**Study population** 15](#_Toc194328060)

[**Figure S2:** Flow chart of cohort enrolment. 15](#_Toc194328061)

[**Table S8:** Proportion of treatment strategies by exposure status for each year of the study period separately. 15](#_Toc194328062)

[**Baseline characteristics** 16](#_Toc194328063)

[**Table S9:** Baseline characteristics of the study population overall and by treatment assignment before and after inverse probability treatment weighting (IPTW). 16](#_Toc194328064)

[**Propensity score distribution before and after weighting** 21](#_Toc194328065)

[**Figure S3:** PS distributions before and after weighting. Abbreviations: PS, propensity score. 21](#_Toc194328066)

[**Distribution of stabilized weights** 22](#_Toc194328067)

[**Figure S4:** Distribution of stabilized weights by treatment group (boxplot) 22](#_Toc194328068)

[**Table S10:** Distribution of stabilized weights overall and by treatment group (table). 22](#_Toc194328069)

[**Primary analysis (intention-to-treat): Subgroup analyses** 23](#_Toc194328070)

[**Table S11:** Number of events, incidence rates, as well as adjusted HRs for the association of DAPT with ticagrelor versus clopidogrel initiation and MACE and major bleeding by subgroups (intention-to-treat). 23](#_Toc194328071)

[**Secondary analysis (per-protocol)** 24](#_Toc194328072)

[**Table S12:** Number of events, incidence rates, as well as crude and adjusted HRs for the association of DAPT with ticagrelor versus clopidogrel initiation and MACE and major bleeding (per-protocol). 24](#_Toc194328073)

[**Quantitative bias analyses (QBA)** 25](#_Toc194328074)

[Rationale 25](#_Toc194328075)

[Methods 25](#_Toc194328076)

[Results and discussion 25](#_Toc194328077)

[**Table S13:** Quantitative bias analyses results based on Schneeweiss et al. (array approach) (1) 26](#_Toc194328078)

[Conclusion 27](#_Toc194328079)

[References (QBA) 27](#_Toc194328080)

# **Study protocol**

## **Protocol of the hypothetical trial and the emulation procedure**

### **Table S1:** Protocol of the hypothetical target trial and the emulation procedure.

| Protocol component | Description of target trial | Description of emulation |
| --- | --- | --- |
| Eligibility criteria | ***Inclusion criteria:***  Patients with a first hospitalization due to an acute coronary syndrome (ACS) between January 1st, 2011 and December 31st, 2021 | ***Inclusion criteria:***  Same as target trial. |
|  | ***Exclusion criteria:*** | ***Exclusion criteria:*** |
|  | Age <75 years | Same as target trial |
|  | Medications: | Medications: |
|  | (i) Use of other P2Y_12_-inhibitor than ticagrelor or clopidogrel prior to hospital admission | (i) dispensation of other P2Y_12_-inhibitor than ticagrelor or clopidogrel prior to index date (defined below) |
|  | (ii) discharged without low-dose aspirin | (ii) no dispensation of low-dose aspirin within 365 days before hospital admission until 14 days after hospital discharge |
|  | (iii) use of oral anticoagulant (OAC) prior to hospital admission | (iii) use of OAC within 365 days prior to index date (defined below) |
| Treatment strategies | 1. Initiation of DAPT with ticagrelor during the ACS hospitalization and continuous use for 365 days  2. Initiation of DAPT with clopidogrel during the ACS hospitalization and continuous use for 365 days | 1. Initiation of DAPT* with ticagrelor within 14 days after hospital discharge** (index date) and continuous use for 365 days  2. Initiation of DAPT* with clopidogrel within 14 days after hospital discharge** (index date) and continuous use for 365 days  *defined as: dispensation of ticagrelor or clopidogrel within 14 days after the hospital discharge date (first ever use) AND dispensation of low-dose aspirin within 180 days prior to or at the day of initiation of ticagrelor or clopidogrel.  **after hospital discharge date because no inpatient drug dispensation recorded in the underlying data source |
| Treatment assignment (also: treatment strategy) | Patients were randomized to either treatment strategy at the index date and were aware of their assigned strategy (no blinding). | We assign individuals to the treatment strategy with which their data are compatible. To emulate randomization, we adjust for the following baseline covariates: age, sex, level of education, percutaneous coronary intervention during ACS hospitalization, type of acute coronary syndrome, medical history (diabetes mellitus, hypertension, atrial fibrillation, other arrythmias, vascular and cerebrovascular diseases (including stroke/TIA/embolism), COPD, cancer, liver disease, heart failure, dyslipidemia, hypothyroidism, intracranial hemorrhage, valvular heart disease, bleeding, anemia, kidney disease, alcoholism, rheumatoid arthritis), medications (β-blockers, calcium channel blockers, diuretics, PPIs, NSAIDs, lipid lowering therapy (e.g., statins), α-blockers, nitrate, antiarrhythmics, diabetes medications, opioids, ACE inhibitors (including combinations), ARBs (including combinations), corticosteroids, antidepressants), and healthcare use in the previous year (number of unique medications, primary care visits, cardiovascular primary care visits, specialist care visits, cardiovascular specialist care visits, hospitalizations, cardiovascular hospitalizations). |
| Outcomes | ***Effectiveness outcome:*** major adverse cardiovascular events (MACE; composite of cardiovascular death, non-fatal myocardial infarction, and non-fatal stroke)  ***Safety outcome:*** major bleeding (hemorrhagic stroke or gastrointestinal bleeding or anemia-related bleeding or other bleeding) | Same as target trial. Cardiovascular mortality is identified from the Swedish death registry; hospitalizations for myocardial infarction, stroke, and major bleeding are identified through ICD-10 codes in the National Patient Register. |
| Causal estimand | ***Primary analysis:*** Intention-to-treat effect (effect of being randomized to treatment)  ***Secondary analysis:*** Per-protocol effect (effect of following the assigned treatment strategy as specified in the protocol) | Same as target trial. |
| Start and end of follow-up | Starts at randomization and ends at the earliest of: outcomes of interest, loss-of-follow-up (stop of participation in target trial), administrative censoring, or 365 days of follow-up. | Starts at DAPT initiation (filled prescription) and ends at the earliest of: outcomes of interest, loss-of-follow-up (migration out of Stockholm region), administrative censoring (Dec 31, 2021), or 365 days of follow-up. |
| Statistical analysis | ***Primary analysis:***  Intention-to-treat analysis | ***Primary analysis:***  Intention-to-treat analysis: Inverse probability of treatment weighting (IPTW) to adjust for baseline covariates; weighted Cox proportional hazard analysis. Plotting weighted cumulative incidence curves using the Aalen-Johansen estimator, which does not overestimate absolute risks in the presence of the competing risk of death. |
|  | ***Secondary analysis:***  Per-protocol analysis | ***Secondary analysis:***  Per-protocol analysis: Effect of following the assigned treatment strategy: Same as primary analysis, but individuals will be censored when no dispensations were received within the 90 days after the estimated end of pill supply from the most recent dispensation |

**Abbreviations:** ACS, acute coronary syndrome; OAC, oral anticoagulant; DAPT, dual antiplatelet therapy; TIA, transient ischemic attack; COPD, chronic obstructive pulmonary disease; PPI, proton-pump inhibitors; NSAID, non-steroidal anti-inflammatory drugs; ACE inhibitors, angiotensin-converting enzyme inhibitors; ARB, angiotensin receptor blocker; MACE, major adverse cardiovascular events; ICD, International Classification of Diseases; IPTW, inverse probability of treatment weighting.

## **Graphical overview of longitudinal study design**


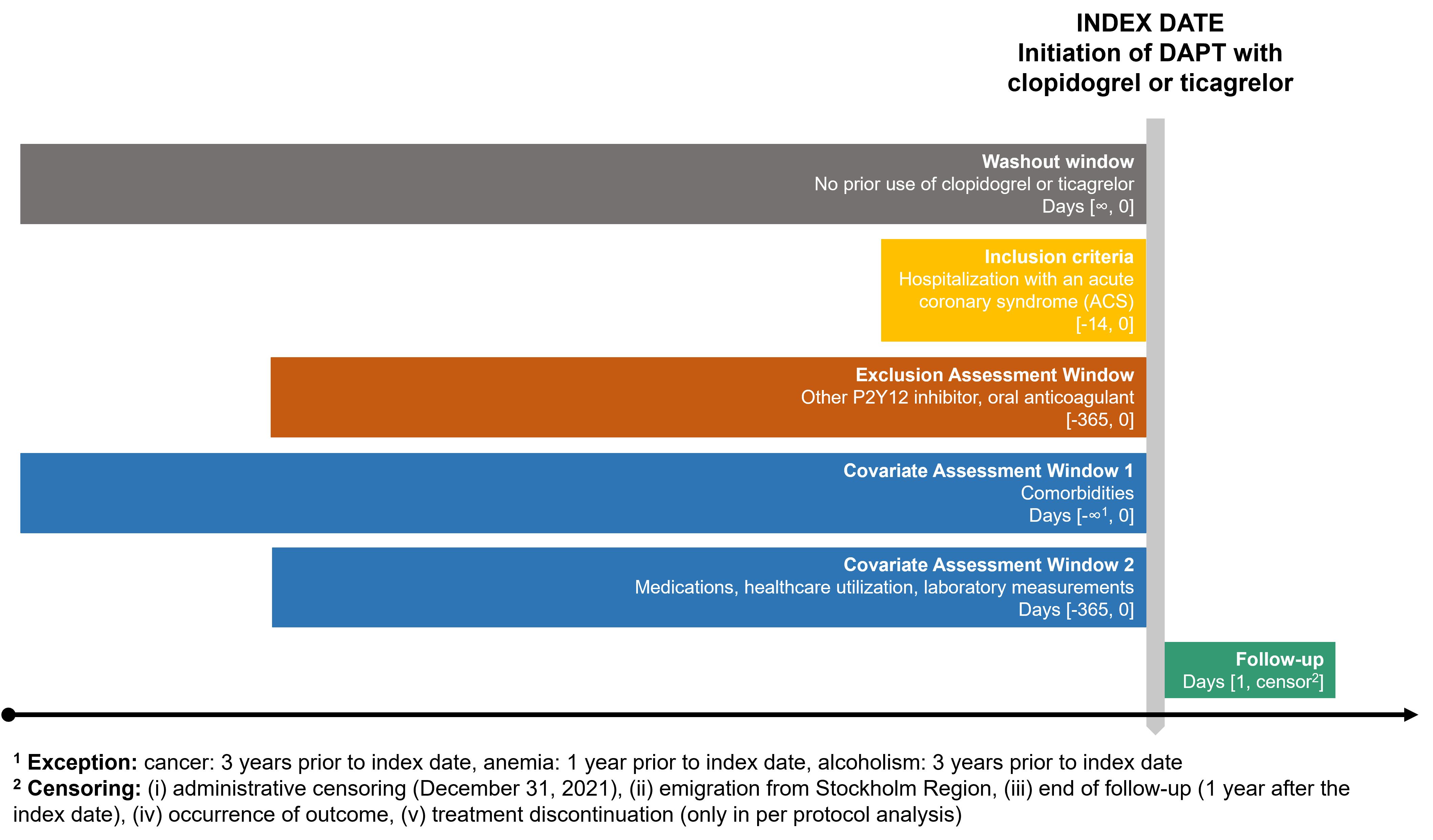


### **Figure S1:** Graphical overview of longitudinal study design.

## **Eligibility criteria**

### **Table S2:** Codes used to identify hospitalizations with an acute coronary syndrome.

| Conditions | ICD-10 code/s | Data source |
| --- | --- | --- |
| Unstable angina | I20.0 (primary position) | National Patient Register (inpatient dataset) |
| Acute myocardial infarction | I21.x (primary position) | National Patient Register (inpatient dataset) |

### **Table S3:** Codes used to identify exclusion criteria.

| Exclusion criteria | Code/s | Data source |
| --- | --- | --- |
| Age <75 years | N/A | Swedish Population Register |
| Death recording during hospitalization | N/A | Swedish Death Registry |
| Prevalent users of other P2Y12 inhibitor prior to index date | ATC: B01AC22, B01AC26, B01AC05 | National Prescribed Drug Register |
| Prevalent users of oral anticoagulants prior to index date | ICD-10: B01AA03, B01AF01, B01AF02, B01AF03, B01AE07 | National Prescribed Drug Register |

## **Treatment strategies**

### **Table S4:** Definitions of treatment assignment.

| Treatment assignment | Description |
| --- | --- |
| DAPT with ticagrelor | Initiation of DAPT* with ticagrelor (ATC code: B01AC06, 60 mg or 90 mg) within 14 days after hospital discharge  *DAPT with ticagrelor is defined as: dispensation of ticagrelor within 14 days after the hospital discharge date (first ever use) AND dispensation of low-dose aspirin (ATC code: B01AC06, dosage: 75 mg) within 180 days prior to or at the day of initiation of ticagrelor. |
| DAPT with clopidogrel | Initiation of DAPT* with clopidogrel (ATC code: B01AC04, dosage: 75 mg) within 14 days after hospital discharge  *DAPT with clopidogrel is defined as: dispensation of clopidogrel within 14 days after the hospital discharge date (first ever use) AND dispensation of low-dose aspirin (ATC code: B01AC06, dosage: 75 mg) within 180 days prior to or at the day of initiation of clopidogrel. |

## **Outcomes & censoring reasons**

### **Table S5:** Definitions of censoring reasons.

| Censoring reason | Definition |
| --- | --- |
| Administrative censoring (end of follow-up) | December 31, 2021 |
| Emigration from Stockholm Region | Emigration out of Stockholm Region after index date (recorded in the Swedish Population Register) |
| End of follow-up | 365 days after the index date |
| Occurrence of outcome | See Supplementary *Table S6* with definitions of outcomes |
| Treatment discontinuation (only in secondary analysis) | No reﬁll of the P2Y12 inhibitor ticagrelor or clopidogrel within a period equal to the days of supply of the last ﬁlled dispensation plus a 90-day grace period. Days of supply for each P2Y12 dispensation were calculated by deﬁned daily doses (DDD)*.  *Number of packages dispensed × number of DDDs dispensed in each package |

### **Table S6:** Definitions of outcomes.

| Outcomes | Code/s | Data source | Definition is based on (PMID) |
| --- | --- | --- | --- |
| Primary effectiveness outcome | | | |
| *MACE (composite outcome)* |  |  |  |
| *Cardiovascular death* | ICD-10: G45‐G46, H34.1, I | Swedish Death Registry | 34196082 |
| *Non-fatal MI* | ICD-10: I21, I22, I23 (primary and secondary position) | National Patient Register (inpatient dataset) | 34196082 |
| *Non-fatal stroke* | ICD-10: G45, G46, H34.1, I60, I61, I63, I64 (primary and secondary position) | National Patient Register (inpatient dataset) | 34196082 |
| Primary safety outcome | | | |
| *Major bleeding (composite outcome)* |  |  |  |
| *Hemorrhagic stroke* | ICD-10: I60-I62 (primary and secondary position) | National Patient Register (inpatient dataset) | 32867508 |
| *Gastrointestinal bleeding* | ICD-10: K226, K250, K252, K254, K256, K260, K262, K264, K266, K270, K272, K274, K276, K280, K282, K284, K286, K290, K625, K920, K921, K922, I850 (primary and secondary position) | National Patient Register (inpatient dataset) | 32867508 |
| *Anemia-related bleeding* | ICD-10: D629, D500 (primary and secondary position) | National Patient Register (inpatient dataset) | 32867508 |
| *Other bleeding* | ICD-10: N421, N938, N939, N950, R041, R042, R048, R049, R210, R319, R210, T810, N501A (primary and secondary position) | National Patient Register (inpatient dataset) | 32867508 |

Abbreviations: MACE, major adverse cardiovascular event; MI, myocardial infarction; ICD-10, International Classification of Diseases 10th Revision

## **Covariates**

### **Table S7:** Definitions of covariates.

| Covariates | Definition / categories | Time window | Source of codes |
| --- | --- | --- | --- |
| Demographics |  |  |  |
| Age | Median (IQR)  Categories: 75-80, 80-85, ≥ 80 | At index date | N/A |
| Female sex | yes/no | - | N/A |
| Highest level of education | Compulsory school, secondary school, university, missing | Latest recording prior to index date | PMID: 30929112 |
| Calendar year of DAPT initiation | - | At index date | N/A |
| DAPT initiation on the day of hospital discharge | Yes/no | Number of days between hospital discharge date and DAPT initiation | N/A |
| Comorbidities |  |  |  |
| Diabetes mellitus | ICD-10: E10, E11, E12, E13, E14 | Any time prior to index date | PMID: [35265981](https://pubmed.ncbi.nlm.nih.gov/35265981) |
| Hypertension | ICD-10: I10, I11, I12, I13, I15 | Any time prior to index date | PMID: [35265981](https://pubmed.ncbi.nlm.nih.gov/35265981) |
| Atrial fibrillation | ICD-10: I48 | Any time prior to index date | PMID: 35861825 |
| Other arrythmia | ICD-10: I44-I47, I49 | Any time prior to index date | PMID: 35861825 |
| Vascular and cerebrovascular diseases | ICD-10: I70, I71, I731, I738, I739, I790, I792, K551, K558, K559, Z958, Z959, G45, G46, I60-69, H340 | Any time prior to index date | PMID: [35265981](https://pubmed.ncbi.nlm.nih.gov/35265981) |
| COPD | ICD-10: J40-47, J60-67, I278, I279, J684, J701, J703 | Any time prior to index date | PMID: [35265981](https://pubmed.ncbi.nlm.nih.gov/35265981) |
| Cancer | ICD-10: C0, C1, C2, C3, C40, C41, C43, C45, C46, C46, C47, C48, C49, C5, C6, C7, C8, C9 | Within 3 years prior to index date | PMID: 32349963 |
| Liver disease | ICD-10: B15-19, C22, D684C, I928B, K70-77, DQ618A, Z944 | Any time prior to index date | PMID: [35265981](https://pubmed.ncbi.nlm.nih.gov/35265981) |
| Heart failure | ICD-10: I43, I50, I099, I110, I130, I132, I255, I420, I425-429, P290 | Any time prior to index date | PMID: [35265981](https://pubmed.ncbi.nlm.nih.gov/35265981) |
| Dyslipidemia | ICD-10: E78 | Any time prior to index date | On own accord (Socialtyrelsen: Search service for health-related classiciations) * |
| Hypothyroidism | ICD-10: E02, E03, E890 | Any time prior to index date | On own accord (Socialtyrelsen: Search service for health-related classiciations) * |
| Intracranial hemorrhage | ICD-10: I60 | Any time prior to index date | On own accord (Socialtyrelsen: Search service for health-related classiciations) * |
| Valvular heart disease | ICD-10: I34, I35, I36, I37, I38, I39 | Any time prior to index date | PMID: 35861825 |
| Bleeding | ICD-10: I60, I61, I62, S064, S065, S066, I850, I983, K25-28 (subcodes 0-2 and 4-6 only), K625, K922, D62 | Any time prior to index date | PMID: [35265981](https://pubmed.ncbi.nlm.nih.gov/35265981) |
| Anemia | ICD-10: D50-59, D60-64 | Within 1 year prior to the index date | PMID: [35265981](https://pubmed.ncbi.nlm.nih.gov/35265981) |
| Kidney disease | ICD-10: E102, E112, E132, E142, I120, M300, M313, M319, M321B, N02-08, N11, N12, N14, N18, N19, N26, N158-160, N162-164, N168, Q612, Q613, Q615, Q619 | Any time prior to index date | PMID: [35265981](https://pubmed.ncbi.nlm.nih.gov/35265981) |
| Alcoholism | ICD-10: E244, F10, G312, G621, G721, I426, K292, K70, K860, O354, P043, Q860, T51, Y90, Y91, Z502, Z714 | Within 3 years prior to index date | PMID: [35265981](https://pubmed.ncbi.nlm.nih.gov/35265981) |
| Previous stroke/TIA/embolism | ICD-10: I63, I64, I679, I693, I694, I698, I67, I69, I74, Z866, Z876, G453, G458, G459 | Any time prior to index date | PMID: [35265981](https://pubmed.ncbi.nlm.nih.gov/35265981) |
| Rheumatoid arthritis | ICD-10: M05, M06, M32-34, M315, M351, M353, M360 | Any time prior to index date | On own accord (Socialtyrelsen: Search service for health-related classiciations) * |
| Medications |  |  |  |
| β-blockers | ATC: C07 | 365 days prior to index date | PMID: 33372009 |
| Calcium channel blockers | ATC: C08 | 365 days prior to index date | PMID: 33372009 |
| Diuretics (loop, thiazide, or potassium-sparing diuretics) | ATC: C03C, C03A, C03D | 365 days prior to index date | PMID: 33372009 |
| NSAIDs | ATC: M01A | 365 days prior to index date | PMID: 31382219 |
| Lipid lowering therapy (all) | ATC: C10 | 365 days prior to index date | PMID: 33246024 |
| Statins | ATC: C10AA | 365 days prior to index date | PMID: [35265981](https://pubmed.ncbi.nlm.nih.gov/35265981) |
| α-blockers | ATC: C02CA, C02LE, G04CA, R03AA, R03CA | 365 days prior to index date | On own accord (ATC/DDD index 2023) ^§^ |
| Nitrate | ATC: C01DA | 365 days prior to index date | On own accord (ATC/DDD index 2023) ^§^ |
| Antiarrhythmics | ATC: C01B | 365 days prior to index date | On own accord (ATC/DDD index 2023) ^§^ |
| Antidiabetic medications | ATC: A10 | 365 days prior to index date | PMID: 33246024 |
| Opioids | ATC: N02A | 365 days prior to index date | On own accord (ATC/DDD index 2023) ^§^ |
| Angiotensin-converting enzyme inhibitors (ACEi) incl. combinations | ATC: C09A, C09B | 365 days prior to index date | PMID: [35265981](https://pubmed.ncbi.nlm.nih.gov/35265981) |
| Angiotensin II receptor blocker (ARB) incl. combinations | ATC: C09C, C09D | 365 days prior to index date | PMID: [35265981](https://pubmed.ncbi.nlm.nih.gov/35265981) |
| Corticosteroids | ATC: H02AA01, H02AA02, H02AA03, H02AB | 365 days prior to index date | PMID: [35265981](https://pubmed.ncbi.nlm.nih.gov/35265981) |
| Antidepressants | ATC: N06A | 365 days prior to index date | PMID: [35265981](https://pubmed.ncbi.nlm.nih.gov/35265981) |
| Healthcare utilization |  |  |  |
| Unique number of medications | Median (IQR) of unique ATC-7 level codes  Categories: 0-3, 4-6, 7-9, ≥ 10 | 365 days prior to index date | N/A |
| Primary care visits | Median (IQR)  Categories: 0, 1-2, 3-4, ≥5 | 365 days prior to index date | N/A |
| Cardiovascular primary care visits | ICD-10: any codes of the I family  Median (IQR)  Categories: 0, 1-2, 3-4, ≥ 5 | 365 days prior to index date | N/A |
| Specialist care visits | Median (IQR)  Categories: 0, 1-2, 3-4, ≥ 5 | 365 days prior to index date | N/A |
| Cardiovascular specialist visits | ICD-10: any codes of the I family  Median (IQR)  Categories: 0, 1-2, 3-4, ≥ 5 | 365 days prior to index date | N/A |
| Hospitalizations (IQR) | Median (IQR)  Categories: 1-2, 3-4, ≥ 5 | 365 days prior to index date | N/A |
| Cardiovascular hospitalizations (IQR) | ICD-10: any codes of the I family  Median (IQR)  Categories: 1-2, 3-4, ≥ 5 | 365 days prior to index date | N/A |
| Laboratory measurements |  |  |  |
| eGFR [ml/min/1.73m^2^] | Exclusion of inpatient values  Categories:  ≤ 60, > 60  Estimation of GFR based on creatinine with CKD-EPI 2009 formula (assuming white ethnicity for everyone) | 365 days prior to index date | N/A |
| Total cholesterol [mmol/l] | Inpatient and outpatient values; categories:  normal (<5), high (≥5), missing | 365 days prior to index date | N/A |
| LDL cholesterol [mmol/l] | Inpatient and outpatient values; categories:  normal (<4), high (≥4), missing | 365 days prior to index date | N/A |
| HDL cholesterol [mmol/l] | Inpatient and outpatient values; categories:  normal (≥1.0 in men, ≥1.2 in women), low (<1.0 in men, <1.2 in women), missing | 365 days prior to index date | N/A |
| Triglycerides [mmol/l] | Inpatient and outpatient values; categories:  normal (<1.69), borderline high (1.69-2.25), high (≥2.26), missing | 365 days prior to index date | N/A |

Abbreviations: N/A, not available; IQR, interquartile range; DAPT, dual antiplatelet therapy; COPD, chronic obstructive pulmonary disease; TIA, transient ischemic attack; ATC, Anatomic Therapeutic Chemical Classification System; NSAIDs, non-steroidal anti-inflammatory drugs; eGFR, estimated glomerular filtration rate; LDL, low-density lipoprotein; HDL, high-density lipoprotein.

* https://klassifikationer.socialstyrelsen.se/

^§^ https://www.whocc.no/atc_ddd_index/

## **Remarks on methodology**

### Weighted Cox proportional hazards regression model

**Description**

The weighted Cox proportional hazards regression model is a statistical method commonly used in observational studies to estimate the effect of a treatment, such as in our target trial emulation study comparing the safety and effectiveness of dual antiplatelet therapy (DAPT) with ticagrelor vs. clopidogrel. In our analysis, we employed inverse probability of treatment weighting (IPTW) to adjust for baseline confounding (in this case, we included 46 potential confounders). IPTW creates a pseudo-population where the distribution of baseline covariates is balanced between the treatment groups, effectively mimicking randomization. The weights are calculated based on the probability of receiving DAPT with ticagrelor or clopidogrel, respectively, conditional on the baseline covariates. This allows to control for confounding variables that could otherwise bias the results. The weighted Cox proportional hazards model then analyzes the time-to-event outcomes (MACE and major bleeding) while accounting for the treatment allocation in the weighted sample.

The purpose of this approach is to provide an accurate estimate of the treatment effect in target trial emulation studies, closely approximating the results of a randomized trial, while benefiting from the ability to reflect data from real-world practice.

**Advantages over multivariable regression (adapted from Fu et al. (1))**

Propensity score (PS) methods (i.e., PS weighting as used in our study) and multivariable regression (i.e., as traditionally performed) have the same potential to control for confounding. However, PS methods have certain advantages compared with traditional multivariable regression.(2)

1. PS methods clearly separate the design and analysis steps: first, confounding is eliminated by balancing the confounders (design step) and afterwards the treatment effect is directly estimated (analysis step).
2. By inspecting the PS distribution, areas of non-overlap between the treated and untreated groups can be identified. Patients in these areas of non-overlap may have an absolute indication or contraindication for treatment (3). In PS matching, these subjects, for whom no comparison can be made, are not matched and hence excluded from the analysis. In PS weighting, these subjects may be identified since they receive very large weights. In our example there was no clear non-overlap of the PS distribution (Figure S3).
3. PS methods may be preferred over multivariable regression analysis when the number of events is low relative to the number of confounders. The reason is that PS methods estimate the relationship between confounders and treatment, whereas multivariable regression estimates the relationship between confounders and outcome. In the case where treatment is common but the number of events is low, there is often enough data to adequately model the relationships between (many) confounders and the treatment in a PS model, but too little data to estimate the relationships between confounders and the outcome in a traditional regression analysis in which the confounders are included as separate covariates (4).
4. In multivariable regression analysis the relationship between each of the separate confounders and the outcome needs to be modeled properly to appropriately adjust for confounding. On the other hand, PS methods do not model the relationships between confounders and outcome but instead model the relationships between confounders and treatment. Whether this was done correctly, i.e. whether it resulted in balance of confounders between treatment groups, can be checked formally and hence provides an opportunity for correction of modeling errors.

**References (remarks on methodology)**

1. Fu Edouard, 2021. Optimal cardiovascular treatment strategies in kidney disease: Causal inference from observational data. <https://hdl.handle.net/1887/3221348> (accessed 21/03/2025).
2. Fu, E. L., Groenwold, R. H. H., Zoccali, C., Jager, K. J., van Diepen, M., & Dekker, F. W. (2019). Merits and caveats of propensity scores to adjust for confounding. *Nephrology, dialysis, transplantation : official publication of the European Dialysis and Transplant Association - European Renal Association*, *34*(10), 1629–1635. https://doi.org/10.1093/ndt/gfy283
3. Uddin MJ, Groenwold RH, Ali MS, de Boer A, Roes KC, Chowdhury MA, et al. Methods to control for unmeasured confounding in pharmacoepidemiology: an overview. Int J Clin Pharm. 2016;38(3):714-23.
4. Groenwold RH, Nelson DB, Nichol KL, Hoes AW, Hak E. Sensitivity analyses to estimate the potential impact of unmeasured confounding in causal research. Int J Epidemiol. 2010;39(1):107-17.

# **Further results**

## **Study population**


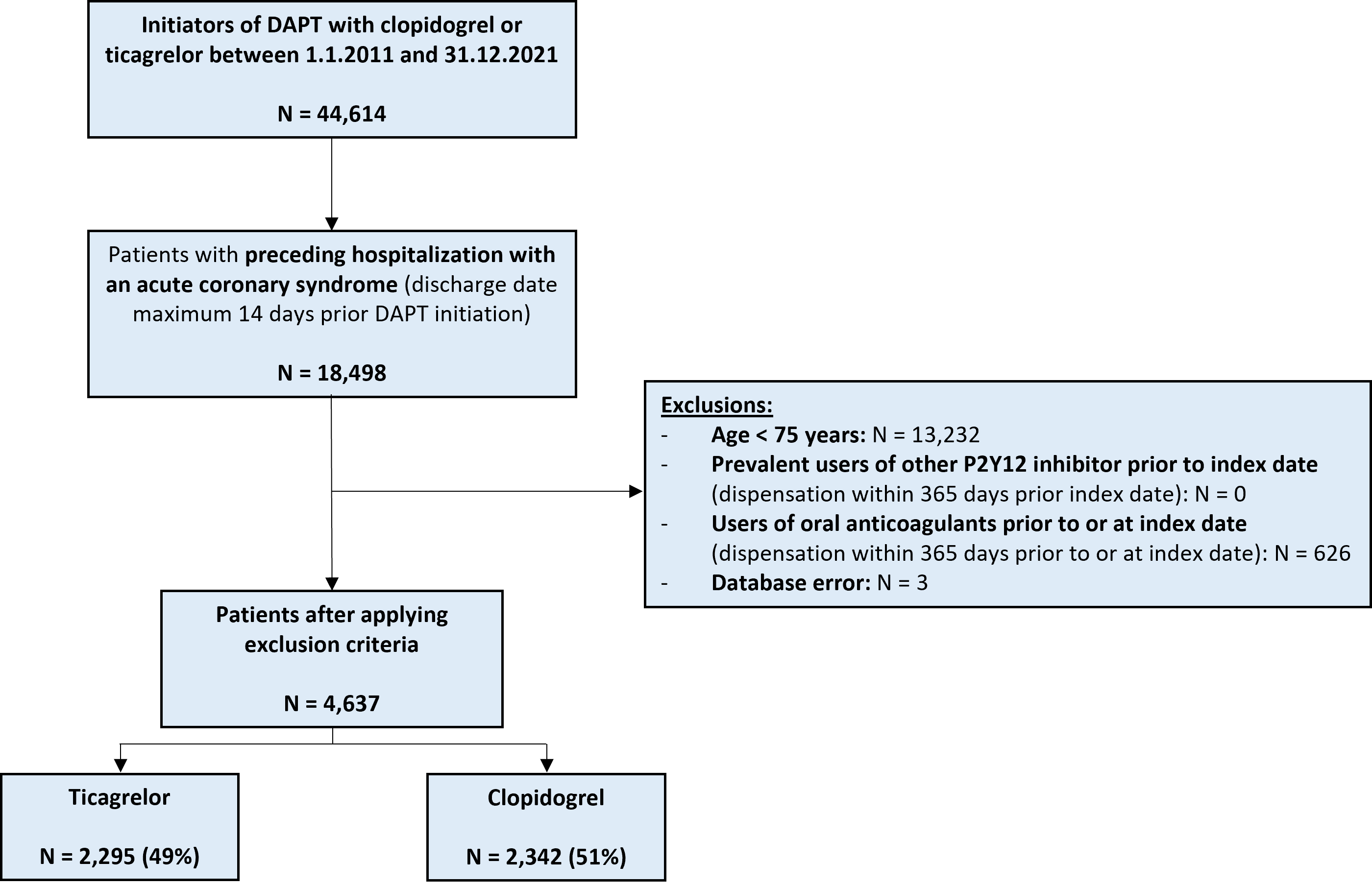


### **Figure S2:** Flow chart of cohort enrolment.

### **Table S8:** Proportion of treatment strategies by exposure status for each year of the study period separately.

| **Year of DAPT initiation** | **DAPT with ticagrelor** | **DAPT with clopidogrel** |
| --- | --- | --- |
| **2011** | 2% | 98% |
| **2012** | 15% | 85% |
| **2013** | 34% | 66% |
| **2014** | 47% | 53% |
| **2015** | 54% | 46% |
| **2016** | 62% | 38% |
| **2017** | 65% | 35% |
| **2018** | 72% | 28% |
| **2019** | 70% | 30% |
| **2020** | 73% | 27% |
| **2021** | 72% | 28% |

## **Baseline characteristics**

### **Table S9:** Baseline characteristics of the study population overall and by treatment assignment before and after inverse probability treatment weighting (IPTW).

|  | Before IPTW | | | | After IPTW | | | |
| --- | --- | --- | --- | --- | --- | --- | --- | --- |
|  | Overall | Ticagrelor | Clopidogrel | SMD^‡^ | Overall | Ticagrelor | Clopidogrel | SMD^‡^ |
| **Number of individuals** | 4637 | 2295 | 2342 |  | 4582 | 2256 | 2326 |  |
| **Demographics** |  |  |  |  |  |  |  |  |
| Age [years], median (IQR) | 81 [77, 85] | 79 [76, 82] | 83 [79, 88] | 0.798 | 80 [77, 85] | 80 [77, 85] | 81 [77, 85] | 0.049 |
| Age category [years] |  |  |  |  |  |  |  |  |
| 75-80 | 1981 (42.7) | 1297 (56.5) | 684 (29.2) | 0.574 | 1990 (43.4) | 999 (44.3) | 991 (42.6) | 0.034 |
| 80-85 | 1384 (29.8) | 725 (31.6) | 659 (28.1) | 0.077 | 1379 (30.1) | 686 (30.4) | 694 (29.8) | 0.013 |
| ≥85 | 1272 (27.4) | 273 (11.9) | 999 (42.7) | 0.737 | 1213 (26.5) | 572 (25.3) | 641 (27.6) | 0.052 |
| Female sex | 2025 (43.7) | 845 (36.8) | 1180 (50.4) | 0.276 | 2006 (43.8) | 989 (43.8) | 1018 (43.8) | 0.001 |
| **Highest level of education achieved *** |  |  |  |  |  |  |  |  |
| Compulsory school | 1482 (32.0) | 616 (26.8) | 866 (37.0) | 0.220 | 1450 (31.7) | 710 (31.5) | 741 (31.8) | 0.006 |
| Secondary school | 1831 (39.5) | 929 (40.5) | 902 (38.5) | 0.041 | 1796 (39.2) | 882 (39.1) | 915 (39.3) | 0.004 |
| University | 1155 (24.9) | 678 (29.5) | 477 (20.4) | 0.211 | 1169 (25.5) | 588 (26.1) | 581 (25.0) | 0.025 |
| **Hospitalization with ACS** |  |  |  |  |  |  |  |  |
| Acute myocardial infarction | 4093 (88.3) | 2015 (87.8) | 2078 (88.7) | 0.029 | 3973 (86.7) | 1942 (86.1) | 2031 (87.3) | 0.036 |
| With PCI | 3190 (68.8) | 2021 (88.1) | 1169 (49.9) | 0.905 | 3194 (69.7) | 1596 (70.7) | 1598 (68.7) | 0.045 |
| **Initiation of DAPT** |  |  |  |  |  |  |  |  |
| Calendar year |  |  |  |  |  |  |  |  |
| 2011-2013 | 1407 (30.3) | 229 (10.0) | 1178 (50.3) | 0.977 | 1477 (32.2) | 251 (11.1) | 1226 (52.7) | 0.997 |
| 2014-2016 | 1267 (27.3) | 686 (29.9) | 581 (24.8) | 0.115 | 1210 (26.4) | 687 (30.4) | 523 (22.5) | 0.180 |
| 2017-2019 | 1214 (26.2) | 836 (36.4) | 378 (16.1) | 0.474 | 1163 (25.4) | 797 (35.3) | 366 (15.7) | 0.462 |
| 2020-2021 | 749 (16.2) | 544 (23.7) | 205 (8.8) | 0.415 | 731 (16.0) | 521 (23.1) | 210 (9.0) | 0.391 |
| Initiation on the day of hospital discharge | 3220 (69.4) | 1726 (75.2) | 1494 (63.8) | 0.250 | 3151 (68.8) | 1598 (70.8) | 1553 (66.7) | 0.089 |
| **Comorbidities ^†^** |  |  |  |  |  |  |  |  |
| Hypertension | 3609 (77.8) | 1760 (76.7) | 1849 (78.9) | 0.054 | 3530 (77.0) | 1732 (76.8) | 1798 (77.3) | 0.013 |
| Diabetes mellitus | 1198 (25.8) | 577 (25.1) | 621 (26.5) | 0.031 | 1189 (26.0) | 587 (26.0) | 602 (25.9) | 0.003 |
| Heart failure | 1297 (28.0) | 455 (19.8) | 842 (36.0) | 0.366 | 1225 (26.7) | 568 (25.2) | 657 (28.3) | 0.070 |
| Valvular heart disease | 385 (8.3) | 120 (5.2) | 265 (11.3) | 0.222 | 378 (8.2) | 184 (8.2) | 194 (8.3) | 0.006 |
| Atrial fibrillation | 484 (10.4) | 110 (4.8) | 374 (16.0) | 0.373 | 520 (11.3) | 265 (11.7) | 255 (11.0) | 0.024 |
| Other arrythmia | 724 (15.6) | 308 (13.4) | 416 (17.8) | 0.120 | 711 (15.5) | 353 (15.6) | 358 (15.4) | 0.006 |
| Vascular and cerebrovascular diseases | 1110 (23.9) | 419 (18.3) | 691 (29.5) | 0.266 | 1115 (24.3) | 534 (23.6) | 581 (25.0) | 0.031 |
| Stroke/TIA/embolism | 631 (13.6) | 230 (10.0) | 401 (17.1) | 0.208 | 593 (12.9) | 275 (12.2) | 318 (13.7) | 0.044 |
| Intracranial hemorrhage | 14 (0.3) | 3 (0.1) | 11 (0.5) | 0.062 | 11 (0.2) | 4 (0.2) | 7 (0.3) | 0.023 |
| Bleeding | 487 (10.5) | 186 (8.1) | 301 (12.9) | 0.155 | 468 (10.2) | 219 (9.7) | 249 (10.7) | 0.033 |
| Anemia | 364 (7.8) | 121 (5.3) | 243 (10.4) | 0.191 | 332 (7.2) | 149 (6.6) | 183 (7.9) | 0.050 |
| Dyslipidemia | 1780 (38.4) | 971 (42.3) | 809 (34.5) | 0.160 | 1810 (39.5) | 888 (39.4) | 921 (39.6) | 0.005 |
| Liver disease | 99 (2.1) | 51 (2.2) | 48 (2.0) | 0.012 | 100.0 (2.2) | 49 (2.2) | 50 (2.2) | 0.002 |
| Kidney disease | 654 (14.1) | 256 (11.2) | 398 (17.0) | 0.169 | 633 (13.8) | 300 (13.3) | 333 (14.3) | 0.030 |
| Alcoholism | 48 (1.0) | 20 (0.9) | 28 (1.2) | 0.032 | 51 (1.1) | 27 (1.2) | 24 (1.0) | 0.018 |
| Cancer | 659 (14.2) | 310 (13.5) | 349 (14.9) | 0.040 | 661 (14.4) | 321 (14.2) | 340 (14.6) | 0.010 |
| Hypothyroidism | 539 (11.6) | 248 (10.8) | 291 (12.4) | 0.051 | 501 (10.9) | 233 (10.3) | 268 (11.5) | 0.039 |
| COPD | 931 (20.1) | 420 (18.3) | 511 (21.8) | 0.088 | 915 (20.0) | 454 (20.1) | 461 (19.8) | 0.008 |
| Rheumatoid arthritis | 440 (9.5) | 187 (8.1) | 253 (10.8) | 0.091 | 408 (8.9) | 193 (8.6) | 215 (9.2) | 0.023 |
| **Co-medications ^§^** |  |  |  |  |  |  |  |  |
| β-blockers | 3928 (84.7) | 1919 (83.6) | 2009 (85.8) | 0.060 | 3927 (85.7) | 1946 (86.3) | 1980 (85.1) | 0.032 |
| ACE inhibitors | 2392 (51.6) | 1215 (52.9) | 1177 (50.3) | 0.054 | 2377 (51.9) | 1167 (51.7) | 1210 (52.0) | 0.006 |
| Angiotensin II receptor blockers | 1549 (33.4) | 842 (36.7) | 707 (30.2) | 0.138 | 1532 (33.4) | 773 (34.3) | 758 (32.6) | 0.036 |
| Calcium channel blockers | 1708 (36.8) | 862 (37.6) | 846 (36.1) | 0.030 | 1746 (38.1) | 869 (38.5) | 877 (37.7) | 0.017 |
| Diuretics | 1583 (34.1) | 596 (26.0) | 987 (42.1) | 0.346 | 1518 (33.1) | 714 (31.6) | 805 (34.6) | 0.063 |
| α-blockers | 397 (8.6) | 224 (9.8) | 173 (7.4) | 0.085 | 438 (9.5) | 228 (10.1) | 210 (9.0) | 0.038 |
| Lipid lowering therapy | 4030 (86.9) | 2167 (94.4) | 1863 (79.5) | 0.453 | 4017 (87.7) | 1994 (88.4) | 2023 (87.0) | 0.042 |
| Of which: statins | 4012 (86.5) | 2160 (94.1) | 1852 (79.1) | 0.453 | 4002 (87.3) | 1987 (88.1) | 2015 (86.6) | 0.044 |
| Nitrate | 3600 (77.6) | 1876 (81.7) | 1724 (73.6) | 0.196 | 3553 (77.5) | 1763 (78.1) | 1791 (77.0) | 0.028 |
| Antiarrhythmics | 19 (0.4) | 7 (0.3) | 12 (0.5) | 0.033 | 18 (0.4) | 7 (0.3) | 11 (0.5) | 0.021 |
| Diabetes medication | 869 (18.7) | 433 (18.9) | 436 (18.6) | 0.006 | 864 (18.8) | 425 (18.8) | 439 (18.9) | 0.001 |
| Proton pump inhibitors | 1919 (41.4) | 910 (39.7) | 1009 (43.1) | 0.070 | 1883 (41.1) | 967 (42.9) | 916 (39.4) | 0.071 |
| NSAIDs | 836 (18.0) | 430 (18.7) | 406 (17.3) | 0.036 | 825 (18.0) | 399 (17.7) | 426 (18.3) | 0.017 |
| Opioids | 921 (19.9) | 373 (16.3) | 548 (23.4) | 0.180 | 927 (20.2) | 449 (19.9) | 478 (20.5) | 0.016 |
| Corticosteroids | 501 (10.8) | 211 (9.2) | 290 (12.4) | 0.103 | 474 (10.3) | 235 (10.4) | 238 (10.2) | 0.006 |
| Antidepressants | 610 (13.2) | 259 (11.3) | 351 (15.0) | 0.110 | 574 (12.5) | 263 (11.6) | 311 (13.4) | 0.053 |
| **Laboratory measurements ^§^** |  |  |  |  |  |  |  |  |
| eGFR ≤ 60 ml/min/1.73 m² * | 1267 (27.3) | 481 (21.0) | 786 (33.6) | 0.286 | 1257 (27.4) | 583 (25.9) | 674 (29.0) | 0.070 |
| High total cholesterol (≥ 5 mmol/L) * | 1791 (38.6) | 987 (43.0) | 804 (34.3) | 0.179 | 1752 (38.2) | 869 (38.5) | 883 (38.0) | 0.010 |
| High LDL cholesterol (≥ 4 mmol/L) * | 593 (12.8) | 336 (14.6) | 257 (11.0) | 0.108 | 602 (13.1) | 306 (13.6) | 297 (12.7) | 0.027 |
| Low HDL cholesterol (< 1.0 mmol/L in men, < 1.2 mmol/L in women) * | 839 (18.1) | 419 (18.3) | 420 (17.9) | 0.010 | 833 (18.2) | 384 (17.0) | 449 (19.3) | 0.060 |
| Triglycerides [mmol/L] * |  |  |  |  |  |  |  |  |
| Borderline high (1.69-2.25) | 567 (12.2) | 333 (14.5) | 234 (10.0) | 0.138 | 591 (12.9) | 328 (14.5) | 264 (11.3) | 0.096 |
| High (≥ 2.26) | 325 (7.0) | 178 (7.8) | 147 (6.3) | 0.059 | 317 (6.9) | 156 (6.9) | 162 (6.9) | <0.001 |
| **Healthcare utilization ^§^** |  |  |  |  |  |  |  |  |
| Unique medications, median (IQR) | 8 [4, 12] | 7 [4, 11] | 9 [5, 13] | 0.353 | 8 [4, 12] | 8 [4, 12] | 8 [5, 12] | 0.007 |
| Unique medications |  |  |  |  |  |  |  |  |
| 0-3 | 867 (18.7) | 550 (24.0) | 317 (13.5) | 0.271 | 842 (18.4) | 435 (19.3) | 407 (17.5) | 0.046 |
| 4-6 | 976 (21.0) | 514 (22.4) | 462 (19.7) | 0.066 | 952 (20.8) | 445 (19.7) | 507 (21.8) | 0.052 |
| 7-9 | 955 (20.6) | 485 (21.1) | 470 (20.1) | 0.025 | 973 (21.2) | 512 (22.7) | 460 (19.8) | 0.071 |
| ≥ 10 | 1839 (39.7) | 746 (32.5) | 1093 (46.7) | 0.293 | 1815 (39.6) | 864 (38.3) | 952 (40.9) | 0.053 |
| Primary care visits, median (IQR) | 2 [1, 5] | 2 [1, 5] | 2 [1, 5] | 0.086 | 2 [1, 5] | 2 [1, 5] | 2 [1, 5] | 0.015 |
| Primary care visits |  |  |  |  |  |  |  |  |
| 0 | 753 (16.2) | 270 (11.8) | 483 (20.6) | 0.241 | 743 (16.2) | 361 (16.0) | 382 (16.4) | 0.011 |
| 1-2 | 1685 (36.3) | 917 (40.0) | 768 (32.8) | 0.150 | 1657 (36.2) | 820 (36.3) | 837 (36.0) | 0.006 |
| 3-4 | 844 (18.2) | 434 (18.9) | 410 (17.5) | 0.036 | 830 (18.1) | 406 (18.0) | 424 (18.2) | 0.005 |
| ≥ 5 | 1355 (29.2) | 674 (29.4) | 681 (29.1) | 0.007 | 1352 (29.5) | 669 (29.7) | 683 (29.3) | 0.009 |
| Cardiovascular primary care visits, median (IQR) | 0 [0, 1] | 0 [0, 1] | 0 [0, 0] | 0.024 | 0 [0, 1] | 0 [0, 1] | 0 [0, 1] | 0.041 |
| Cardiovascular primary care visits |  |  |  |  |  |  |  |  |
| 0 | 3450 (74.4) | 1625 (70.8) | 1825 (77.9) | 0.163 | 3400 (74.2) | 1673 (74.2) | 1727 (74.3) | 0.002 |
| 1-2 | 1081 (23.3) | 634 (27.6) | 447 (19.1) | 0.202 | 1053 (23.0) | 519 (23.0) | 534 (22.9) | 0.002 |
| 3-4 | 78 (1.7) | 26 (1.1) | 52 (2.2) | 0.086 | 82 (1.8) | 30 (1.3) | 53 (2.3) | 0.075 |
| ≥ 5 | 28 (0.6) | 10 (0.4) | 18 (0.8) | 0.052 | 47 (1.0) | 34 (1.5) | 13 (0.5) | 0.094 |
| Specialist care visits, median (IQR) | 4 [2, 8] | 4 [2, 7] | 4 [2, 8] | 0.074 | 4 [2, 8] | 4 [2, 8] | 4 [2, 8] | 0.019 |
| Specialist care visits |  |  |  |  |  |  |  |  |
| 0 | 476 (10.3) | 240 (10.5) | 236 (10.1) | 0.013 | 482 (10.5) | 244 (10.8) | 238 (10.2) | 0.020 |
| 1-2 | 1117 (24.1) | 610 (26.6) | 507 (21.6) | 0.117 | 1124 (24.5) | 547 (24.2) | 578 (24.8) | 0.014 |
| 3-4 | 964 (20.8) | 492 (21.4) | 472 (20.2) | 0.030 | 962 (21.0) | 506 (22.4) | 456 (19.6) | 0.069 |
| ≥ 5 | 2080 (44.9) | 953 (41.5) | 1127 (48.1) | 0.133 | 2014 (43.9) | 959 (42.5) | 1055 (45.3) | 0.056 |
| Cardiovascular specialist visits, median (IQR) | 0 [0, 1] | 0 [0, 1] | 0 [0, 1] | 0.064 | 0 [0, 1] | 0 [0, 1] | 0 [0, 1] | 0.017 |
| Cardiovascular specialist care visits |  |  |  |  |  |  |  |  |
| 0 | 2696 (58.1) | 1375 (59.9) | 1321 (56.4) | 0.071 | 2670 (58.3) | 1325 (58.7) | 1346 (57.8) | 0.018 |
| 1-2 | 1458 (31.4) | 710 (30.9) | 748 (31.9) | 0.022 | 1435 (31.3) | 706 (31.3) | 729 (31.3) | <0.001 |
| 3-4 | 344 (7.4) | 153 (6.7) | 191 (8.2) | 0.057 | 338 (7.4) | 166 (7.3) | 172 (7.4) | 0.004 |
| ≥ 5 | 139 (3.0) | 57 (2.5) | 82 (3.5) | 0.059 | 139 (3.0) | 59 (2.6) | 80 (3.4) | 0.047 |
| Hospitalizations, median (IQR) | 1 [1, 2] | 1 [1, 2] | 2 [1, 2] | 0.273 | 1 [1, 2] | 1 [1, 2] | 1 [1, 2] | 0.016 |
| Hospitalizations |  |  |  |  |  |  |  |  |
| 1-2 | 3731 (80.5) | 1951 (85.0) | 1780 (76.0) | 0.229 | 3660 (79.9) | 1815 (80.4) | 1845 (79.3) | 0.027 |
| 3-4 | 710 (15.3) | 283 (12.3) | 427 (18.2) | 0.165 | 690 (15.1) | 327 (14.5) | 363 (15.6) | 0.031 |
| ≥ 5 | 196 (4.2) | 61 (2.7) | 135 (5.8) | 0.154 | 232 (5.1) | 115 (5.1) | 118 (5.1) | <0.001 |
| Cardiovascular hospitalizations, median (IQR) | 1 [1, 2] | 1 [1, 2] | 1 [1, 2] | 0.145 | 1 [1, 2] | 1 [1, 2] | 1 [1, 2] | 0.005 |
| Cardiovascular hospitalizations |  |  |  |  |  |  |  |  |
| 1-2 | 4242 (91.5) | 2117 (92.2) | 2125 (90.7) | 0.054 | 4141 (90.4) | 2034 (90.1) | 2108 (90.6) | 0.017 |
| 3-4 | 362 (7.8) | 168 (7.3) | 194 (8.3) | 0.037 | 398 (8.7) | 200 (8.9) | 198 (8.5) | 0.014 |
| ≥ 5 | 33 (0.7) | 10 (0.4) | 23 (1.0) | 0.072 | 43 (0.9) | 22 (1.0) | 20 (0.9) | 0.010 |

Values are n (%), unless otherwise indicated.

Abbreviations: IPTW, inverse probability of treatment weighting; SMD, standardized mean difference; IQR, interquartile range; DAPT, dual antiplatelet therapy; ACS, acute coronary syndrome; PCI, percutaneous coronary intervention; TIA, transient ischemic attack; COPD, chronic obstructive pulmonary disease; ACE, angiotensin-converting enzyme; NSAIDs, non-steroidal anti-inflammatory drugs; eGFR, estimated glomerular filtration rate; LDL, low-density lipoprotein; HDL, high-density lipoprotein.

*highest level of education achieved, eGFR, total cholesterol, LDL cholesterol, HDL cholesterol, and triglycerides were missing in 3.6%, 26.5%, 14.4%, 17.5%, 17.4%, and 16.8%.

^†^Comorbidities: Any time prior to index date (exceptions: cancer: 3 years prior to index date, anemia: 1 year prior to index date, alcoholism: 3 years prior to index date).

^§^Medications, healthcare utilization, laboratory measurements: 1 year prior to index date.

^‡^A standardized mean difference (SMD) above 0.1 indicates meaningful imbalance between groups.

## **Propensity score distribution before and after weighting**

| 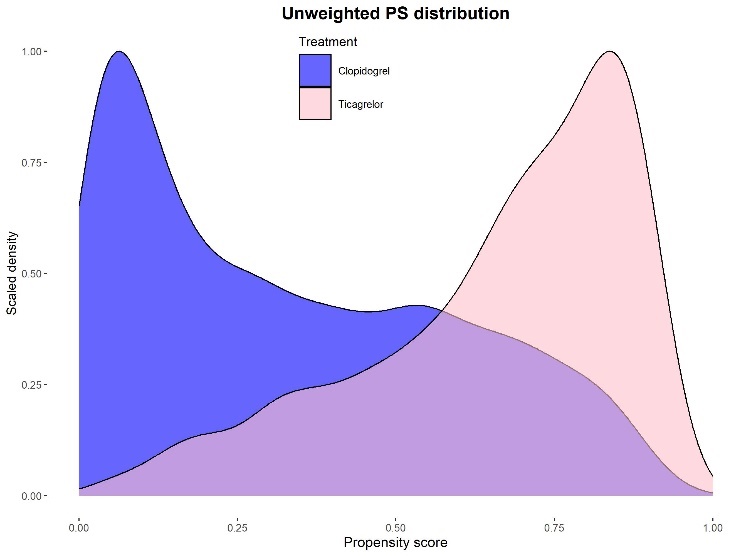 | 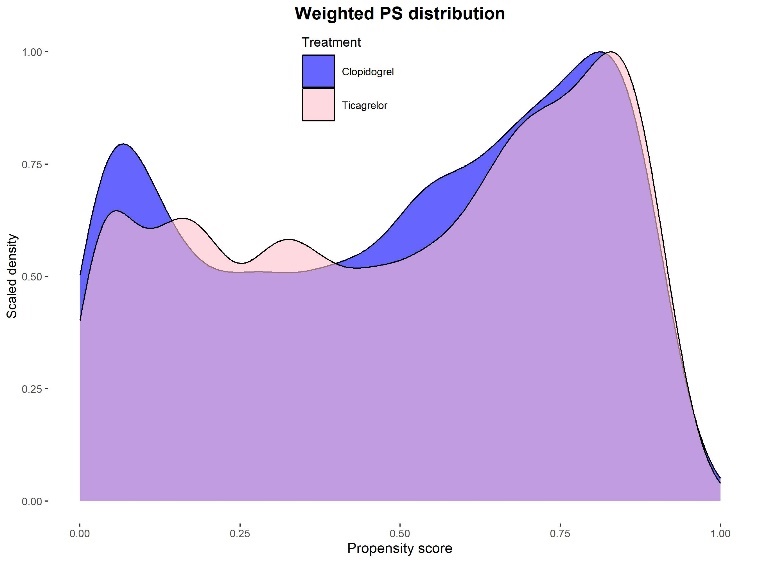 |
| --- | --- |

### **Figure S3:** PS distributions before and after weighting. Abbreviations: PS, propensity score.

## **Distribution of stabilized weights**


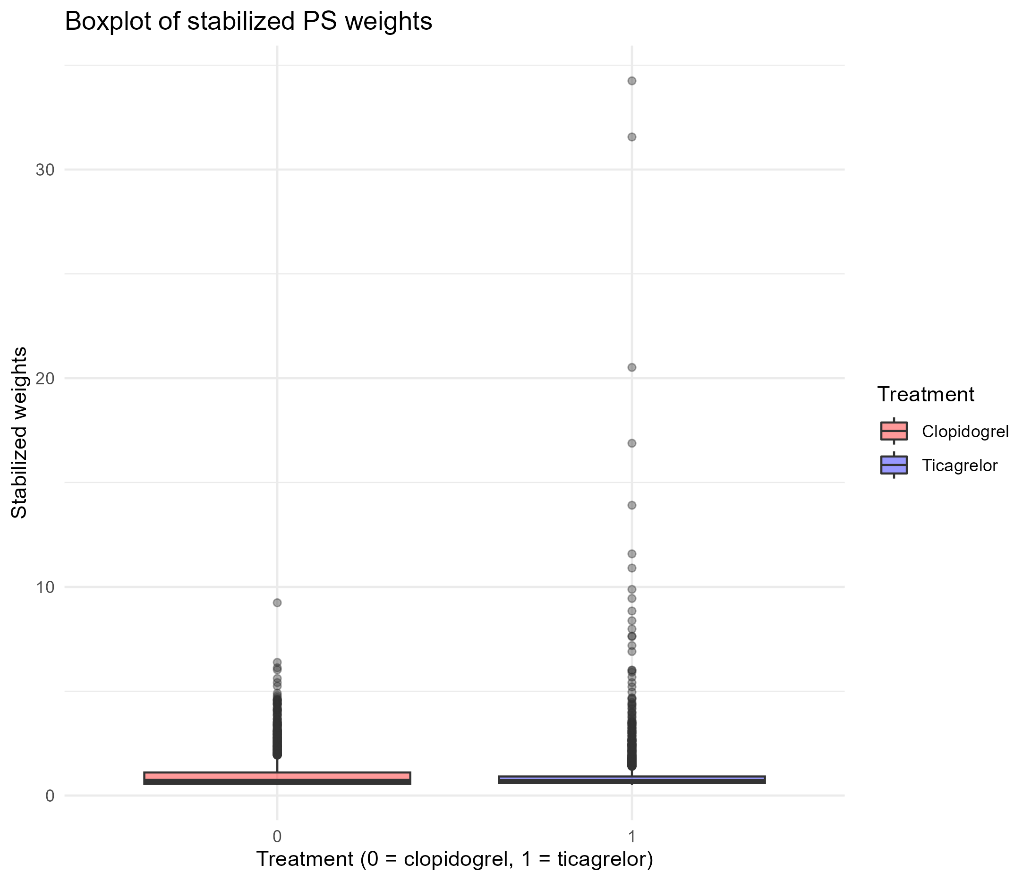


### **Figure S4:** Distribution of stabilized weights by treatment group (boxplot)

### **Table S10:** Distribution of stabilized weights overall and by treatment group (table).

|  | **Minimum** | **1st quartile** | **Median** | **Mean** | **3rd quartile** | **Maximum** |
| --- | --- | --- | --- | --- | --- | --- |
| **Overall** | 0.5055 | 0.5798 | 0.6935 | 0.9882 | 1.0105 | 34.2507 |
| **Clopidogrel** | 0.5055 | 0.5543 | 0.6982 | 0.9932 | 1.1042 | 9.2389 |
| **Ticagrelor** | 0.5163 | 0.5954 | 0.6909 | 0.9830 | 0.9135 | 34.2507 |

## **Primary analysis (intention-to-treat): Subgroup analyses**

### **Table S11:** Number of events, incidence rates, as well as adjusted HRs for the association of DAPT with ticagrelor versus clopidogrel initiation and MACE and major bleeding by subgroups (intention-to-treat).

|  | No. of patients^a^ | No. of events^a^ | IR per 100 py (95% CI)^a^ | IPTW adjusted HR (95% CI)^b^ | P-value for interaction |
| --- | --- | --- | --- | --- | --- |
| MACE |  |  |  |  |  |
| Female |  |  |  |  | 0.741 |
| Yes | 2025 | 74 | 10.1 (7.9-12.6) | 0.68 (0.44-1.03) |  |
| No | 2612 | 130 | 10.5 (8.7-12.4) | 0.74 (0.55-0.98) |  |
| Decreased kidney function |  |  |  |  | 0.176 |
| eGFR < 60 ml/min/1.73 m² | 1267 | 51 | 12.5 (9.3-16.4) | 0.52 (0.32-0.85) |  |
| eGFR ≥ 60 ml/min/1.73 m² | 2139 | 93 | 9.4 (7.6-11.5) | 0.80 (0.55-1.15) |  |
| PCI |  |  |  |  | 0.533 |
| Yes | 3190 | 175 | 10.0 (8.6-11.6) | 0.81 (0.63-1.05) |  |
| No | 1447 | 29 | 12.5 (8.4-18.0) | 0.67 (0.39-1.15) |  |
| Major bleeding |  |  |  |  |  |
| Female |  |  |  |  | 0.957 |
| Yes | 2025 | 30 | 3.9 (2.7-5.6) | 0.97 (0.56-1.66) |  |
| No | 2612 | 75 | 5.9 (4.6-7.4) | 0.95 (0.60-1.50) |  |
| Decreased kidney function |  |  |  |  | 0.514 |
| eGFR < 60 ml/min/1.73 m² | 1267 | 22 | 5.1 (3.2-7.8) | 0.69 (0.38-1.27) |  |
| eGFR ≥ 60 ml/min/1.73 m² | 2139 | 59 | 5.8 (4.4-7.5) | 0.89 (0.56-1.42) |  |
| PCI |  |  |  |  | 0.949 |
| Yes | 3190 | 95 | 5.3 (4.3-6.5) | 0.89 (0.63-1.26) |  |
| No | 1447 | 10 | 4.1 (2.0-7.6) | 0.92 (0.35-2.46) |  |

**Abbreviations:** no., number; py, person-years; IR, incidence rate; HR, hazard ratio; IPTW, inverse probability of treatment weighting; CI, confidence interval; MACE, major adverse cardiovascular event; eGFR, estimated glomerular filtration rate; PCI, percutaneous coronary intervention. **^a^**Number of patients, number of events, py, and IRs were calculated in the unweighted population. **^b^**Analyses were adjusted through inverse probability of treatment weighting for age, sex, level of education, percutaneous coronary intervention during ACS hospitalization, type of acute coronary syndrome, medical history (diabetes mellitus, hypertension, atrial fibrillation, other arrythmias, vascular and cerebrovascular diseases (including stroke/TIA/embolism), COPD, cancer, liver disease, heart failure, dyslipidemia, hypothyroidism, intracranial hemorrhage, valvular heart disease, bleeding, anemia, kidney disease, alcoholism, rheumatoid arthritis), medications (β-blockers, calcium channel blockers, diuretics, PPIs, NSAIDs, lipid lowering therapy (e.g., statins), α-blockers, nitrate, antiarrhythmics, diabetes medications, opioids, ACE inhibitors (including combinations), ARBs (including combinations), corticosteroids, antidepressants), and healthcare use in the previous year (number of unique medications, primary care visits, cardiovascular primary care visits, specialist care visits, cardiovascular specialist care visits, hospitalizations, cardiovascular hospitalizations).

## **Secondary analysis (per-protocol)**

### **Table S12:** Number of events, incidence rates, as well as crude and adjusted HRs for the association of DAPT with ticagrelor versus clopidogrel initiation and MACE and major bleeding (per-protocol).

| Outcomes | Exposure | No. of patients^a^ | No. of events^a^ | Median follow-up [IQR], days | IR per 100 py (95% CI)^a^ | Crude HR (95% CI) | IPTW adjusted HR (95% CI)^b^ |
| --- | --- | --- | --- | --- | --- | --- | --- |
| MACE | Clopidogrel | 2342 | 341 | 190 [190-280] | 26.4  (23.7-29.4) | Reference | Reference |
|  | Ticagrelor | 2295 | 172 | 238 [140-280] | 12.6  (10.7-14.6) | 0.51  (0.43-0.61) | 0.79  (0.59-1.07) |
| Major  bleeding | Clopidogrel | 2342 | 86 | 190 [190-280] | 6.4  (5.1-7.9) | Reference | Reference |
|  | Ticagrelor | 2295 | 83 | 238 [140-280] | 5.9  (4.7-7.3) | 0.95  (0.70-1.29) | 0.96  (0.64-1.45) |

Abbreviations: no., number; py, person-years; IR, incidence rate; HR, hazard ratio; IPTW, inverse probability of treatment weighting; MACE, major adverse cardiovascular event.

^a^Number of patients, number of events, py, and IRs were calculated in the unweighted population.

^b^Analyses were adjusted through inverse probability of treatment weighting for age, sex, level of education, percutaneous coronary intervention during ACS hospitalization, type of acute coronary syndrome, medical history (diabetes mellitus, hypertension, atrial fibrillation, other arrythmias, vascular and cerebrovascular diseases (including stroke/TIA/embolism), COPD, cancer, liver disease, heart failure, dyslipidemia, hypothyroidism, intracranial hemorrhage, valvular heart disease, bleeding, anemia, kidney disease, alcoholism, rheumatoid arthritis), medications (β-blockers, calcium channel blockers, diuretics, PPIs, NSAIDs, lipid lowering therapy (e.g., statins), α-blockers, nitrate, antiarrhythmics, diabetes medications, opioids, ACE inhibitors (including combinations), ARBs (including combinations), corticosteroids, antidepressants), and healthcare use in the previous year (number of unique medications, primary care visits, cardiovascular primary care visits, specialist care visits, cardiovascular specialist care visits, hospitalizations, cardiovascular hospitalizations).

## **Quantitative bias analyses (QBA)**

## Rationale

Despite adjusting for 46 baseline confounders via inverse probability of treatment weighting (IPTW), it is possible that residual confounding remains due to unmeasured confounders (e.g., frailty).

## Methods

To assess the potential impact of unmeasured confounding, we conducted a quantitative bias analysis (QBA) using the array approach, as described by Schneeweiss et al. (1). The array approach is a systematic method to evaluate how a range of confounder prevalence and strength of confounder-outcome association combinations would affect the observed treatment effect. This allows for a transparent exploration of residual confounding across various parameter constellations.

Among possible unmeasured confounders, we believe frailty as being the strongest factor to potentially alter our results. Based on literature, frailty is prevalent in approximately 40% of patients aged ≥80 years with acute coronary syndrome (2). Given that patients receiving clopidogrel tend to be sicker in our study, we assumed frailty prevalence to be higher in this group (50%) and lower in the ticagrelor group (30%), while maintaining an overall average prevalence of 40%.

Based on these plausible assumptions we performed a QBA separately for the outcomes MACE and major bleeding.

## Results and discussion

**MACE**

Our QBA for MACE demonstrated that even if frail patients had a 2-fold increased risk of developing MACE compared to non-frail patients (RR_CD_ = 2.0 in *Table S13*), the observed treatment benefit of ticagrelor (HR 0.73 [ARR in *Table S13*], 95% CI 0.56-0.95) would still hold. Specifically, as shown in *Table S13*, the HR (RR_adjusted_ in *Table S13*) would shift to 0.84, but the protective effect of ticagrelor would not be fully explained away.

A 2-fold increased risk of MACE in frail vs. non-frail patients is likely an overestimate due to the following two reasons:

1. A study published in the *European Heart Journal* (3) found that frailty was associated with a 1.77-fold increased risk of MACE (HR 1.77, 95% CI 1.53-2.06) after adjusting for multiple confounders, including age, sex, race/ethnicity, census division, residence, income, body mass index, smoking status, diabetes, hypertension, dependency, and number of chronic diseases. Given this evidence, the true association between frailty and MACE is likely weaker than 2-fold, meaning that our assumption of a 2-fold increased risk is a conservative scenario—even in this exaggerated case, frailty alone would not negate the observed treatment benefit of ticagrelor.
2. One can argue that a more than 2-fold increased risk of MACE in frail vs. non-frail patients is still plausible (i.e., not too far from the 1.77-fold increased risk of MACE observed in the *European Heart Journal* study), which would lead to the conclusion that ticagrelor is not superior in terms of MACE compared to clopidogrel. However, given that we already adjusted our analysis for 46 potential confounders (of note, several of them proxies for frailty), it is unlikely that the strength of the association between frailty and MACE is more than 2-fold.

**Major bleeding**

The QBA in terms of major bleeding demonstrated that even if frail patients were 2 times more likely to develop the outcome major bleeding compared to non-frail patients (RR_CD_ = 2.0 in *Table S13*), the observed lack of difference between ticagrelor and clopidogrel (HR 0.89 [ARR in *Table S13*], 95% CI 0.63-1.27) would remain unchanged. Specifically, as shown in *Table S13*, the HR (RR_adjusted_ in *Table S13*) would shift to 1.03, still showing no difference between ticagrelor and clopidogrel with regards to major bleeding.

A more than 2.0-fold increased risk of bleeding in frail vs. non-frail patients is likely an overestimate given the following information obtained from the literature:

A systematic review and meta-analysis assessing the risk of frailty on bleeding in patients with acute myocardial infarction (4) found that frailty was associated with a 1.34-fold increased risk of bleeding (pooled HR from 7 studies = 1.34, 95% CI 1.12-1.59). Given this evidence, the true association between frailty and bleeding is likely weaker than 2.0-fold, meaning that our assumption of a 2.0-fold increased risk is a conservative scenario—even in this exaggerated case, frailty alone cannot alter our conclusion that there is no significant difference between ticagrelor and clopidogrel.

### **Table S13:** Quantitative bias analyses results based on Schneeweiss et al. (array approach) (1)

| **MACE** | | | | | |
| --- | --- | --- | --- | --- | --- |
| **ARR^1^** (fix) | **RR_CD_^2^** | **P_C1_^3^** | **P_C0_^4^** (fix) | **RR_adjusted_^5^** | **% Bias** |
| 0.73 | 5.5 | 0.3 | 0.5 | 1.01 | -27.69 |
| 0.73 | 5.0 | 0.3 | 0.5 | 1.00 | -26.67 |
| 0.73 | 4.5 | 0.3 | 0.5 | 0.98 | -25.45 |
| 0.73 | 4.0 | 0.3 | 0.5 | 0.96 | -24.00 |
| 0.73 | 3.5 | 0.3 | 0.5 | 0.94 | -22.22 |
| 0.73 | 3.0 | 0.3 | 0.5 | 0.91 | -20.00 |
| 0.73 | 2.5 | 0.3 | 0.5 | 0.88 | -17.14 |
| ***0.73*** | ***2.0*** | ***0.3*** | ***0.5*** | ***0.84*** | ***-13.33*** |
| 0.73 | 1.5 | 0.3 | 0.5 | 0.79 | -8.00 |
| 0.73 | 1.0 | 0.3 | 0.5 | 0.73 | 0.00 |
| **Major bleeding** | | | | | |
| **ARR^1^** (fix) | **RR_CD_^2^** | **P_C1_^3^** | **P_C0_^4^** (fix) | **RR_adjusted_^5^** | **% Bias** |
| 0.89 | 5.5 | 0.3 | 0.5 | 1.23 | -27.69 |
| 0.89 | 5.0 | 0.3 | 0.5 | 1.21 | -26.67 |
| 0.89 | 4.5 | 0.3 | 0.5 | 1.19 | -25.45 |
| 0.89 | 4.0 | 0.3 | 0.5 | 1.17 | -24.00 |
| 0.89 | 3.5 | 0.3 | 0.5 | 1.14 | -22.22 |
| 0.89 | 3.0 | 0.3 | 0.5 | 1.11 | -20.00 |
| 0.89 | 2.5 | 0.3 | 0.5 | 1.07 | -17.14 |
| ***0.89*** | ***2.0*** | ***0.3*** | ***0.5*** | ***1.03*** | ***-13.33*** |
| 0.89 | 1.5 | 0.3 | 0.5 | 0.97 | -8.00 |
| 0.89 | 1.0 | 0.3 | 0.5 | 0.89 | 0.00 |

^1^ ARR: Apparent (or observed) exposure relative risk (in our study: HR)

^2^ RR_CD_: Association between confounder (frailty) and disease outcome (MACE respectively major bleeding)

^3^ P_c1_: Prevalence of confounder (frailty) in the exposed (ticagrelor group)

^4^ P_c0_: Prevalence of confounder (frailty) in the unexposed (clopidogrel group)

^5^ RR_adjusted_: “True” or fully adjusted exposure relative risk

## Conclusion

In conclusion, these findings indicate that additional adjustment for frailty is unlikely to alter our conclusions regarding MACE and major bleeding, given that we already adjusted for 46 covariates, many of which serve as proxies for frailty (e.g., age, comorbidities).

## References (QBA)

1. Schneeweiss S. (2006). Sensitivity analysis and external adjustment for unmeasured confounders in epidemiologic database studies of therapeutics. *Pharmacoepidemiology and drug safety*, *15*(5), 291–303. <https://doi.org/10.1002/pds.1200>
2. Llaó, I., Ariza-Solé, A., Sanchis, J., Alegre, O., López-Palop, R., Formiga, F., Marín, F., Vidán, M. T., Martínez-Sellés, M., Sionis, A., Vives-Borrás, M., Gómez-Hospital, J. A., Gómez-Lara, J., Roura, G., Díez-Villanueva, P., Núñez-Gil, I., Maristany, J., Asmarats, L., Bueno, H., Abu-Assi, E., … Cequier, À. (2018). Invasive strategy and frailty in very elderly patients with acute coronary syndromes. *EuroIntervention : journal of EuroPCR in collaboration with the Working Group on Interventional Cardiology of the European Society of Cardiology*, *14*(3), e336–e342. <https://doi.org/10.4244/EIJ-D-18-00099>
3. Damluji, A. A., Chung, S. E., Xue, Q. L., Hasan, R. K., Moscucci, M., Forman, D. E., Bandeen-Roche, K., Batchelor, W., Walston, J. D., Resar, J. R., & Gerstenblith, G. (2021). Frailty and cardiovascular outcomes in the National Health and Aging Trends Study. *European heart journal*, *42*(37), 3856–3865. <https://doi.org/10.1093/eurheartj/ehab468>
4. Putthapiban P, Vutthikraivit W, Rattanawong P, Sukhumthammarat W, Kanjanahattakij N, Kewcharoen J, Amanullah A. Association of frailty with all-cause mortality and bleeding among elderly patients with acute myocardial infarction: a systematic review and meta-analysis. J Geriatr Cardiol. 2020 May;17(5):270-278. doi: 10.11909/j.issn.1671-5411.2020.05.006. PMID: 32547610; PMCID: PMC7276305.
